# Chemical insights into the atmospheric oxidation of thiophene by hydroperoxyl radical

**DOI:** 10.1038/s41598-021-92221-z

**Published:** 2021-06-22

**Authors:** Maryam Seyed Sharifi, Hamed Douroudgari, Morteza Vahedpour

**Affiliations:** Department of Chemistry, University of Zanjan, PO Box 38791-45371, Zanjan, Iran

**Keywords:** Atmospheric chemistry, Computational chemistry

## Abstract

The reaction mechanisms and kinetics of thiophene oxidation reactions initiated by hydroperoxyl radical, and decomposition of the related intermediates and complexes, have been considered herein by using high-level DFT and ab initio calculations. The main energetic parameters of all stationary points of the suggested potential energy surfaces have been computed at the BD(T) and CCSD(T) methods, based on the geometries optimized at the B3LYP/6-311 + g(d,p) level of theory. Rate constants of bimolecular reactions (high-pressure limit rate constants) at temperatures from 300 to 3000 K for the first steps of the title reaction have been obtained through the conventional transition state theory (TST), while the pressure dependent rate constants and the rate constants of the second and other steps have been calculated employing the Rice–Ramsperger–Kassel–Marcus/Master equation (RRKM/ME). The results show that the rate constants of addition to α and β carbons have positive temperature dependence and negative pressure dependence. It is found that the additions of HO_2_ to the α and β carbons of thiophene in the initial steps of the title reaction are the most favored pathways. Also, the addition to the sulfur atom has a minor contribution. But, all efforts for simulating hydrogen abstraction reactions have been unsuccessful. In this complex oxidation reaction, about 12 different products are obtained, including important isomers such as thiophene-epoxide, thiophene-ol, thiophene-oxide, oxathiane, and thiophenone. The calculated total rate constants for generation of all minimum stationary points show that the addition reactions to the α and β carbons are the fastest among all at temperatures below 1000 K, while the proposed multi-step parallel reactions are more competitive at temperatures above 1200 K. Furthermore, important inter-and intra-molecular interactions for some species have been investigated by two well-known quantum chemistry method, the NBO and AIM analyses. Thermochemical properties such as free energy, enthalpy, internal energy, and entropy for thiophene and hydroperoxyl radical and related species in the simulated reactions have been predicted using a combination of the B3LYP and BD(T) methods.

## Introduction

Heteroaromatic compounds have been detected in the air analysis of large cities. Thiophene and its derivatives are one of the most important heteroaromatics. They are emitted into the atmosphere through different processes, such as the incomplete combustion of fossil fuels, products of oil distillation, gas injection into coal, burning plants and other biomasses, volcanic emissions, and also it has been detected over seaweed fields^1–5^. In asphaltene structure, thiophene and substituted thiophenes are also two main subunits^6^. Moreover, the emission of products yielding from the combustion of sulfur compounds into the atmosphere increases the production of acid rain, ozone, and smog, leading to device corrosion and environmental hazards^7^. The permissible level of sulfur compounds in transport fuels has been drastically reduced by governments to combat air pollution^8^. But, according to the aerosol samples of different experiments, the highest concentrations of thiophene derivatives were found in industrial sites^9^.

To remove sulfur compounds from fuels and petroleum products, several methods have been recommended, leading to improve the quality of fuel combustion. Conventional hydrodesulfurization (HDS) is the most popular one but it is not effective for eliminating heterocyclic sulfur compounds such as dibenzothiophene (DBT) and its derivatives^10^. Other methods that are used for removing sulfur compounds are selective adsorption, biodesulphurization, oxidation/extraction (oxidative desulphurization), extraction with ionic liquids, photochemical reactions, and liquid–liquid extraction, TiO_2−x_ modified Fe-based catalysts, etc.^11–20^.

Oxidation of thiophene and its derivatives are of great interest for researchers in the fields of atmospheric and combustion chemistry due to developing industry by using fossil fuels. Therefore numerous experimental and computational studies have been performed on the oxidation of thiophene-based compounds up to now^12,21–30^. To explore mechanistic features of the thiophene and NO_3_ reaction, Zhang et al. did a computational study in the gas phase. Two main mechanisms were direct hydrogen abstraction and addition–elimination^23^. Song et al. investigated the mechanism of thiophene and methylthiophene in reaction with molecular oxygen on both triplet and singlet potential energy surfaces. They showed that the reaction with singlet molecular oxygen is more favorable for oxidizing thiophene and 2-methylthiophene. They also understood that alkylation of the thiophene ring helps to facilitate the oxidation reaction due to lowering the barrier height of the addition pathway. Shiroudi and Deleuze studied the atmospheric reaction of thiophene initiated by hydroxyl radicals using density functional methods. Their computed rate constants are in good agreement with experimental results that show the OH addition to the α carbon is the most efficient process kinetically^26^.

HO_x_ radicals (OH and HO_2_) are significant oxidizing agents in both combustion and atmospheric chemistry. They produce aerosols through the formation of compounds having low volatility, such as sulfuric acid, nitric acid, and some organics^31^. The oxidation of VOCs with HO_2_ has received a lot of attention from experimental and theoretical aspects^32–34^. Also, hydroperoxyl radical plays a pivotal role in ozone production and degradation in different layers of the atmosphere, namely degradation is observed in the catalytic cycle of stratospheric ozone, and the production is seen in reaction with NO_x_ in the troposphere^35^.

Since sulfur-based pollutants in the atmosphere have a pivotal role in aerosol formation in the urban and industrial zones. And, as we know, the atmospheric oxidation of aerosols affects the removal of toxic compounds. In this article, we will discuss the connection between theoretical viewpoints and the atmospheric relevance of thiophene plus hydroperoxyl radical reaction in the gas phase. In this regard, we will answer this question “how thiophene is degraded by HO_2_ in atmospheric conditions?” To have a deep insight into the question, different parallel reactions will be designed using the high level of quantum chemical methods. Therefore, a multiwell-multichannel reaction is expected for the reaction. After tracing the PES, it will be answered the other main question “which reactions are the main reaction pathways in the atmospheric condition for C_4_H_4_S + HO_2_ reaction?” To find the answers to the questions exactly using theoretical calculations, a validated methodology is required. Thus, we will use the very popular density functional method, B3LYP, for finding all possible paths that have been examined successfully through different multiwell-multichannel reactions in the same conditions^36–44^. Also, to attain exact energies, the main effect of singly, doubly and primitive triply transitions on the proposed PES will be computed by the coupled-cluster theory (CCSD(T) along with the very perfect one, the Brueckner Doubles BD(T). Furthermore, for better clarity of the answers, the important atmospheric variables such as temperature and altitude (pressure) will be investigated by rate constant calculations on the title reaction. Therefore, bimolecular rate constants at high-pressure limit will be calculated by using the transition state (TST) theory for all minima structures produced after prereactive collision complex. And also the pressure-dependent rate constants will be computed by the Rice–Ramsperger–Kassel–Marcus (RRKM) theory in the fall of regime region. However, the stability of obtained minima stationary points will be evaluated thermodynamically using the BD(T) (energies) + B3LYP (thermodynamic corrections) level at 298 K. In addition, the nature of the formation and cleavage of bonds along with significant interactions in the course of the reaction will be determined by the topological analysis of the electronic charge density and the natural bond orbital analysis. The current study opens new insights into the interpretation of the reactivity of aromatic compounds in the gas phase and into designing future experiments and dynamic studies.

## Computational methods

Previously, Zhang et al.^23^ studied the structures of the reaction of thiophene plus NO_3_ by using the popular hybrid density functional method, B3LYP, in conjunction with a Pople triple zeta basis set. Also, the atmospheric reaction of thiophene with OH radical is considered by the CBS-QB3 method and geometry optimization in this method is also executed at the B3LYP method. Therefore, the geometries of reactants, products, intermediates, and transition states in the C_4_H_4_S + HO_2_ reaction were optimized at the B3LYP^45^ method along with the 6–311 + g(d,p) basis set, by using the Gaussian 09 suite of programs^46^. To understand whether the transition states are connected properly to the corresponding reactants and intermediates or products along with the desired reaction paths, the intrinsic reaction coordinate (IRC)^47^ calculations were carried out at the same level of geometry optimization. In addition, the obtained geometries at the B3LYP method are used to achieve more accurate energies by single-point calculations at the CCSD(T)/CC-PV(T + d)Z level^48^ and also to get precise partition function by frequency calculations for dynamic calculations on the potential energy surfaces (PES). The T1 diagnostic values are computed at the CCSD(T)/CC-PV(T + d)Z level for each species in doublet PES. These values are necessary to get information about the multi-reference based methods requirement^49^. Based on the calculated T1 diagnostic values, it can be concluded that the energies of stationary points calculated by single-reference-based methods are acceptable or no, and also to give a qualitative assessment for the importance of non-dynamic correction (or adequacy of the single determinant methods). If the T1 diagnostic value for an open-shell (closed-shell) species is about 0.045 (0.02) or less, the single reference method is sufficient for predicting the reliable energies^50,51^. To describe open-shell systems precisely, another important parameter is the spin contamination value. The amount of spin contamination for our studied species in the doublet state is around 0.75. Accordingly, the single point energy calculations were computed for all stationary points at the BD(T) method as a high-level computational method^52^.

The nature of interactions in all stationary points is identified by the atom in molecule theory (AIM) through the bonds and rings critical points, and the natural bond orbital (NBO) analysis as well^53,54^.

## Results and discussion

### The gas-phase reaction of thiophene with HO_2_

**Figure 1:**
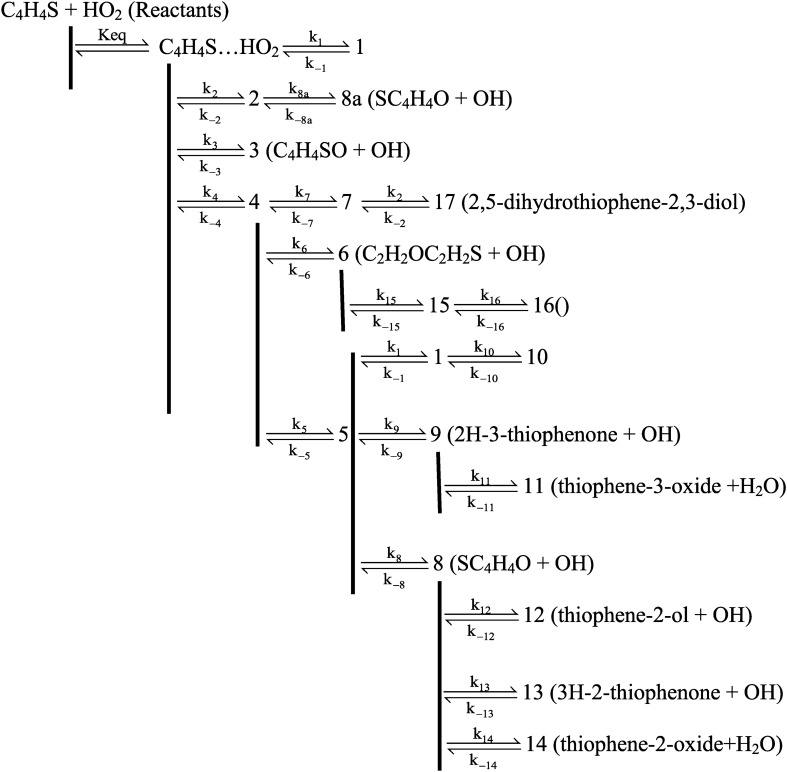
The schematic representation of all the reaction pathways suggested for thiophene plus HO_2_ reaction.

**Figure 2:**
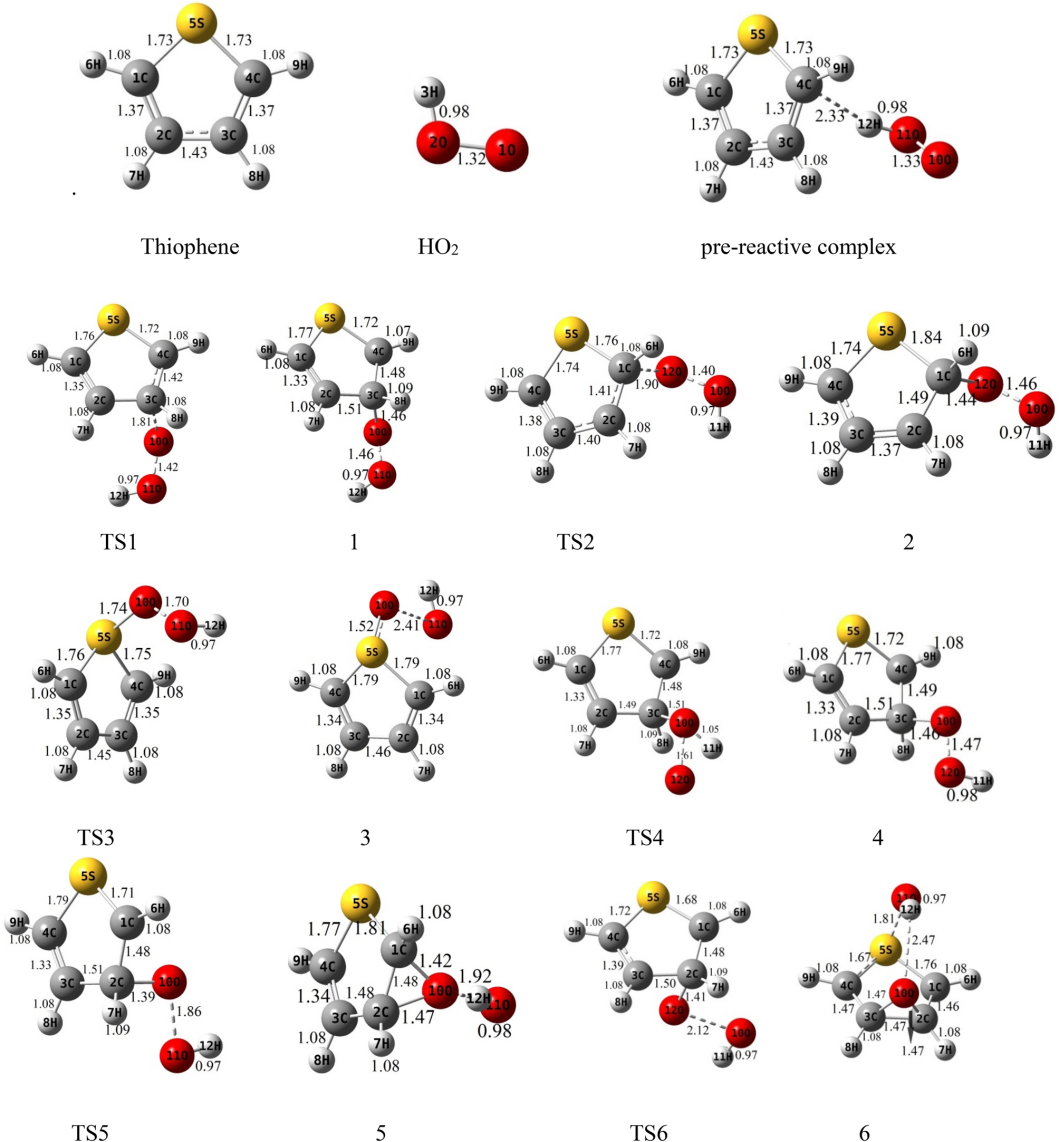

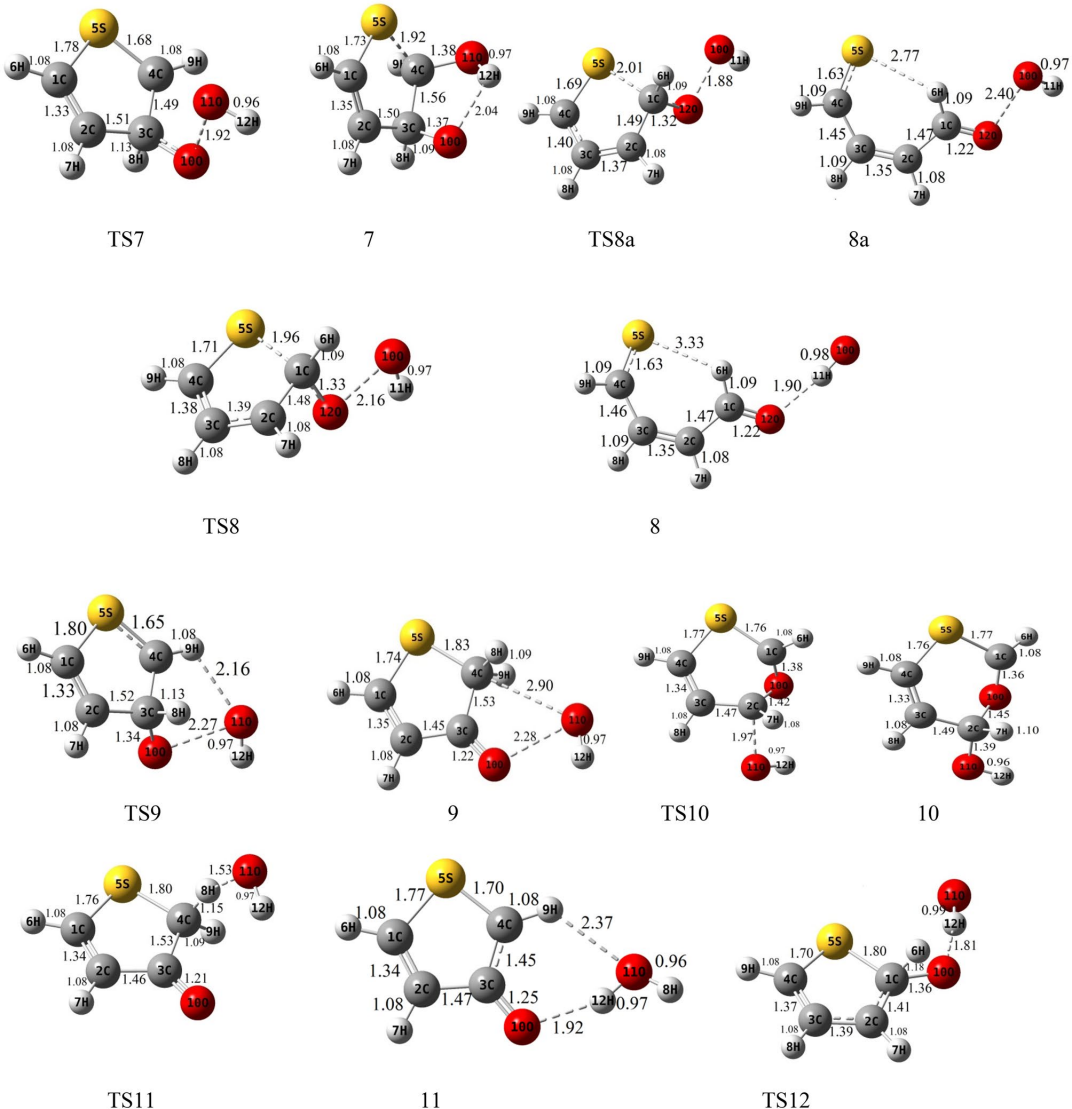

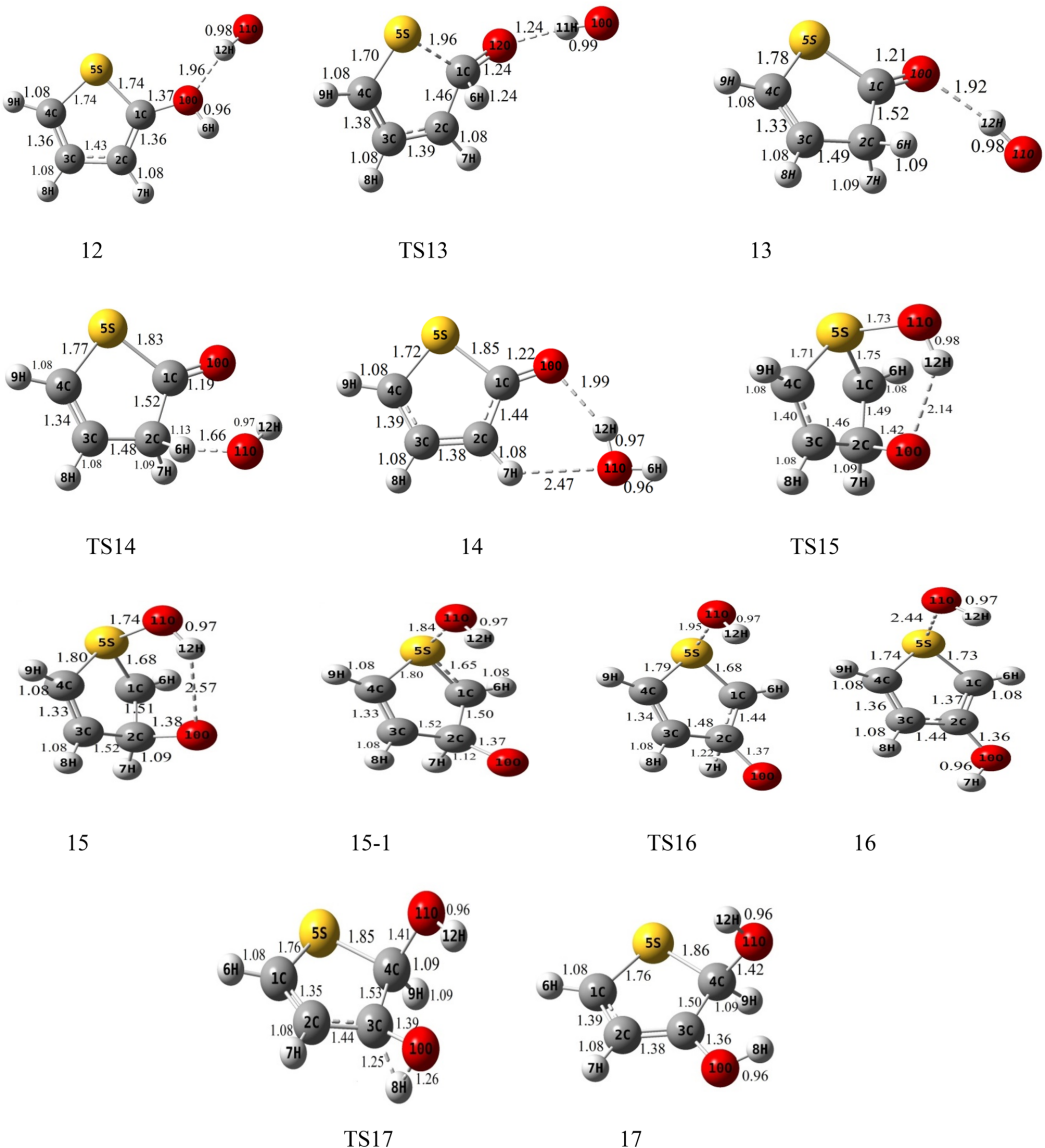

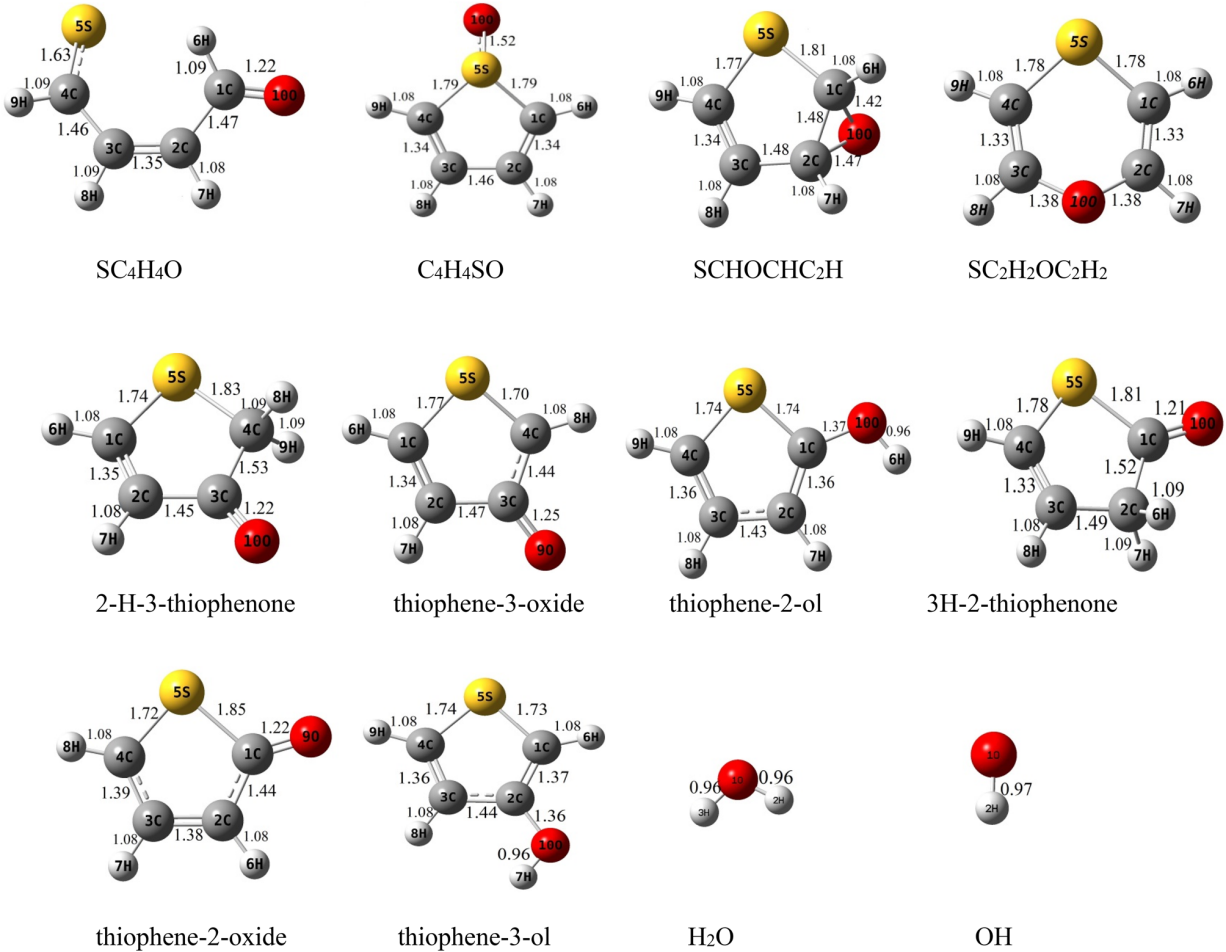
Geometries of reactants, transition states, intermediates, and products optimized at the B3lyp/6-311 + g(d,p) level (bond distances are in angstrom).

**Figure 3:**
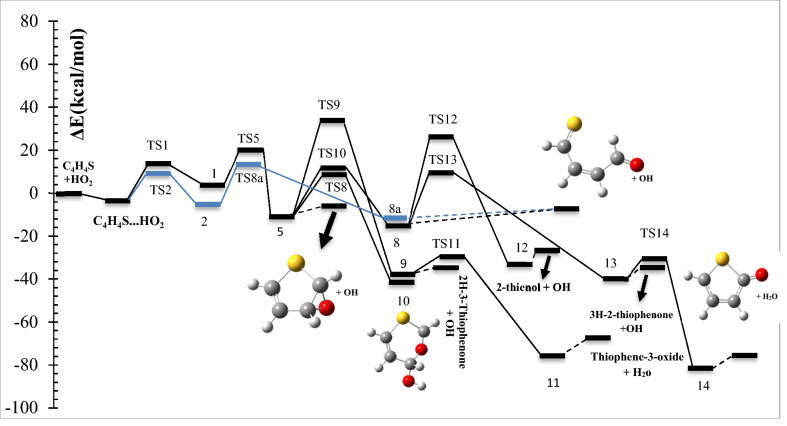
Potential energy profile for the thiophene + HO_2_ reaction including the addition of terminal oxygen of hydroperoxyl radical to α-C (blue) and β-C (black) carbons and reactions of obtained products sketched at the BD(T) 6-31 + g(d,p) level.

**Figure 4:**
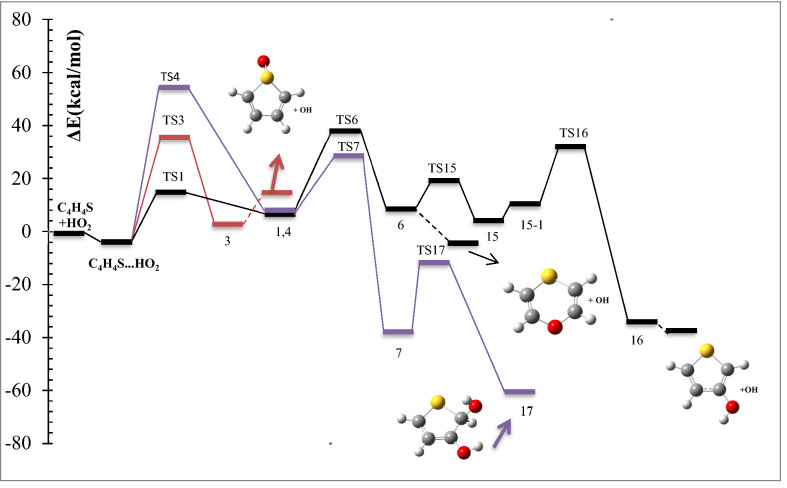
Potential energy profile of the thiophene + HO_2_ reaction including the addition of middle oxygen of HO_2_ radical to β-C (purple) and sulfur (red) atoms, and reactions of obtained products drawn at the BD(T)/6-31 + g(d,p) level.

The schematic representation of all reaction pathways is depicted in Fig. 1. The optimized geometries at the B3LYP/6-311 + g(d,p) level are shown in Fig. 2. The profiles of the potential energy surface (PES) calculated at the BD(T)/631 + g(d,p) level are sketched in Figs. 3 and 4. The relative energies of all species calculated compared to the original reactants are listed in Table 1. Also, the obtained T1 diagnostic values of all interacted molecules at the CCSD(T) method are reported in Table 1. Based on the reported data in Table 1, it may be seen that T1 values are higher than the threshold value for TS6 and TS7. So, for eliminating the doubt of the CCSD(T) energies as a single reference method, we used a high level of calculations by using Brueckner double excitation (BD) method, including primitive triple transitions BD(T). The 6–31 + g(d,p) basis set is selected for the BD(T) calculations because of having a very large computational cost, particularly for cyclic compounds. The output of the BD(T) calculations revealed that changes in the energy of motioned transition states are about 5 kcal mol^−1^ in comparison with the corresponding energies at the CCSD(T) method. Since the BD(T) method has more accurate energies than the CCSD(T) method, throughout the study the energies of that method are used for PES description. The expected values for spin <S^2^> calculated at the B3LYP/6-311 + g(d,p) levels are listed in supplementary materials (Supplementary Table S1). The amounts of standard thermodynamic functions (the internal energies, enthalpies, and Gibbs free energies and entropies) at 298.15 K are calculated at the BD(T)/631 + g(d,p) level and presented in Table 2. Also, the thermodynamic data at the B3LYP/6-311 + g(d,p) level are presented in Supplementary Tables S2 and S3. The details of AIM results for all complexes are reported in Supplementary Table S4.

**Table 1:** Calculated relative energies (in kcal mol^−1^) of the reactants, products, intermediates, and transition states at three different methods.

Stationary points	B3LYP/6-311 + g(d,p)	CCSD (T)/CC-PV(T + d)Z	BD(T)/6-31 + g(d,p)	T1^a^
Thiophene + HO_2_	0	0	0	–
C_4_H_4_S…HO_2_	0.68	− 6.39	− 6.64	0.021
1	15.89	5.76	4.47	0.023
2	5.95	− 2.58	− 4.98	0.025
3	18.62	5.49	9.33	0.017
4	15.94	5.98	4.64	0.023
5	1.69	− 8.06	− 12.57	0.016
6	24.59	9.37	12.71	0.023
7	− 30.84	− 37.49	− 40.94	0.029
8a	− 7.68	− 7.15	− 14.16	0.020
8	− 9.33	− 7.96	− 17.28	0.031
9	− 34.86	− 37.11	− 41.27	0.022
10	− 34.07	− 45.11	− 45.67	0.023
11	− 69.17	− 73.96	− 75.29	0.032
12	− 26.60	− 33.72	− 36.03	0.025
13	− 35.69	− 41.33	− 44.58	0.015
14	− 78.90	− 81.65	− 83.52	0.029
15	16.71	5.42	7.27	0.032
15-1	17.86	12.92	15.16	0.137
16	− 29.89	− 32.56	− 34.09	0.031
17	− 55.53	− 64.75	− 65.01	0.024
SCHOCHC_2_H_2_ + OH	1.64	− 1.77	− 5.13	–
SC_4_H_4_O + OH	− 9.34	− 4.56	− 10.55	–
C_4_H_4_SO + OH	19.26	10.24	15.02	–
SC_2_H_2_OC_2_H_2_ + OH	− 3.7	− 5.15	− 7.01	–
2H-3-Thiophenone + OH	− 34.47	− 32.98	− 36.23	–
Thiophene-3-oxide + H_2_O	− 67.07	− 64.93	− 66.53	–
Thiophene-2-ol + OH	− 28.04	− 29.40	− 30.29	–
3H-2-Thiophenone + OH	− 35.62	− 34.67	− 37.91	–
Thiophene-2-oxide + H_2_O	− 79.11	− 74.08	− 75.88	–
Thiophene-3-ol + OH	− 29.85	− 31.45	− 31.83	–
TS1	21.72	17.59	14.69	0.035
TS2	16.51	14.49	10.61	0.035
TS3	38.41	35.24	32.80	0.038
TS4	63.36	56.39	54.22	0.034
TS5	25.81	25.96	18.36	0.044
TS6	41.61	39.40	37.15	0.087
TS7	30.25	33.05	27.81	0.067
TS8a	14.16	22.34	12.42	0.053
TS8	14.84	21.59	10.96	0.031
TS9	31.25	36.94	29.47	0.033
TS10	19.51	15.32	12.44	0.034
TS11	− 32.73	− 32.49	− 34.84	0.022
TS12	32.17	38.59	29.85	0.048
TS13	10.65	13.24	11.03	0.022
TS14	− 33.96	− 35.50	− 38.24	0.023
TS15	28.70	14.52	18.18	0.031
TS16	35.59	35.09	35.92	0.039
TS17	− 6.16	− 10.67	− 12.21	0.027

^a^Computed T1 diagnostic values at the CCSD (T)/CC-PV(T + d)Z level.

**Table 2:** The thermodynamic parameters of all stationery points computed at room temperature using the BD(T)/6-31 + g(d,p) energies and the B3LYP/6-311 + g(d,p) corrections (unit of all numbers is kcal mol^−1^).

Species	△E°	△H°	△G°	− T△S°
Thiophene + HO_2_	0.00	0.00	0.00	0.00
C_4_H_4_S…HO_2_	− 5.36	− 5.96	3.23	9.19
1	6.25	5.66	16.58	10.93
2	− 3.14	− 3.73	7.42	11.15
3	9.23	8.63	18.62	9.99
4	6.39	5.79	16.63	10.83
5	− 11.43	− 12.02	− 2.73	9.29
6	13.55	12.95	24.90	11.95
7	− 37.96	− 38.56	− 26.41	12.15
8a	− 13.23	− 13.83	− 7.37	6.46
8	− 16.30	− 16.89	− 10.27	6.63
9	− 39.96	− 40.55	− 31.43	9.12
10	− 42.73	− 43.32	− 31.44	11.89
11	− 73.32	− 73.91	− 64.84	9.08
12	− 34.69	− 35.28	− 27.58	7.70
13	− 43.80	− 44.39	− 36.05	8.35
14	− 81.52	− 82.11	− 73.63	8.48
15	8.66	8.06	19.68	11.61
16	− 32.63	− 33.22	− 24.22	9.00
17	− 62.05	− 62.64	− 50.96	11.68
TS1	15.64	15.05	26.07	11.02
TS2	11.57	10.98	21.99	11.02
TS3	32.78	32.19	42.36	10.17
TS4	53.17	52.58	63.81	11.23
TS5	18.58	17.99	28.21	10.22
TS6	36.41	35.81	45.30	9.48
TS7	27.16	26.57	37.20	10.63
TS8a	12.71	11.52	22.54	11.01
TS8	11.04	10.44	20.20	9.75
TS9	28.38	27.79	38.51	10.72
TS10	12.57	11.98	23.47	11.49
TS11	− 35.70	− 36.29	− 25.92	10.37
TS12	27.60	27.01	36.00	8.98
TS13	9.08	8.48	17.33	8.84
TS14	− 38.74	− 39.33	− 29.06	10.27
TS15	18.46	17.87	30.36	12.50
TS16	33.78	33.19	44.19	11.00
TS17	− 12.74	− 13.34	− 1.48	11.86
SCHOCHC_2_H_2_ + OH	− 6.22	− 6.22	− 3.46	2.76
SC_4_H_4_O + OH	− 11.83	− 11.83	− 12.19	− 0.36
C_4_H_4_SO + OH	13.29	13.29	14.99	1.70
SC_2_H_2_OC_2_H_2_ + OH	− 7.97	− 7.97	− 6.32	1.65
2H-3-Thiophenone + OH	− 37.16	− 37.16	− 35.55	1.61
Thiophene-3-oxide + H_2_O	− 67.21	− 67.21	− 66.24	0.97
Thiophene-2-ol + OH	− 31.18	− 31.18	− 29.64	1.54
3H-2-Thiophenone + OH	− 38.98	− 38.98	− 37.43	1.55
Thiophene-2-oxide + H_2_O	− 76.39	− 76.39	− 75.51	0.88
Thiophene-3-ol + OH	− 32.73	− 32.73	− 31.29	1.44

All complexes and transition states, and some radical products of the current study are open-shell species (the doublet state). So, the spin contamination values were checked after each calculation. As shown in Table S1, the expected values of ⟨S^2^⟩_obs_ before spin annihilation are in a range from 0.7522 (OH) to 0.9657 (TS6). After spin annihilation, these values were decreased to 0.7500–0.7538, which have only a 0.51% difference from the actual value (0.7500) expected for a doublet state. This allows the calculations to be taken into account acceptable^55^.

### Addition–elimination pathways

The oxidation reaction between thiophene and hydroperoxyl radical begins via the formation of a van der Waals interaction (*ρ*(*r*_*LCP*_) = 0.01604 e bohr^−3^ and *∇*^2^*ρ*(*r*_*LCP*_) = 0.0414 e bohr^−5^) between the α carbon of thiophene and the H atom of HO_2_, leading to produce a pre-reactive complex (C_4_H_4_O…HO_2_). The reaction continues if the necessary activation energy is supplied for the next steps. In the atmosphere, the sun’s radiation is a source for supplying that energy. Our simulated reaction proceeds by the addition of hydroperoxyl radical to α-C (C1or C4) or β-C (C2 or C3). However, the electrophilic addition of HO_2_ to the S atom could occur with a small contribution. The hydrogen abstraction from the thiophene ring is also examined. The efforts failed to find probable pathways.

#### HO_2_ addition to α-C and S

The terminal oxygen of HO_2_ attacks the α-C and compound 2 is formed. This process occurs via TS2 with relative energy of 10.61 kcal mol^−1^ and an imaginary frequency of 521i cm^−1^ in the reaction coordinate. The distance between C1 and O12 in TS2 is about 1.90 Å and is 1.44 Å in complex 2. The adduct 2 is 4.98 kcal mol^−1^ more stable than the initial reactants. AIM analysis of 2 indicates the presence of a line critical point, LCP^56^, located between O10 and O12 with the electronic charge density *ρ*(*r*_*LCP*_) = 0.2648 e bohr^−3^ and the Laplacian of electronic charge density*∇*^2^*ρ*(*r*_*LCP*_) = 0.0895 e bohr^−5^. The small value and positive sign of the computed Laplacian confirms a weak covalent bond between two oxygen atoms. Then, the reaction continues with TS8a, and the post-reactive complex 8a is produced with relative energy of − 14.16 kcal mol^−1^. Finally, OH and a linear product (SC_4_H_4_O) is produced by getting only the energy of 3.25 kcal mol^−1^. This path is shown with a blue dash line in Fig. 2. We will discuss the generation of these products in another pathway (see “Rearrangement pathways of 5”).

The electrophilic attack of HO_2_ on the S atom is sketched by the red line in Fig. 4. This process occurs through TS3, leading to generate the van der Waals complex product 3 (*ρ*(*r*_*LCP*_) = 0.0252 e bohr^−3^ and *∇*^2^*ρ*(*r*_*LCP*_) = 0.1146 e bohr^−5^). The distance of the S–O bond in TS3 is 1.74 Å and changes to 1.52 Å in 3. The post-reactive complex 3 lays 9.33 kcal mol^−1^ above the initial reactants due to the bond among non-bonding electrons of the sulfur atom and free radical electron of HO_2_ and decreasing the ring aromaticity. Breaking the van der Waals interaction between two oxygen atoms generates the final adducts, OH and thiophene-1-oxide. The relative energy and the standard enthalpy of these products are 14.66 kcal mol^−1^ and 13.29 kcal mol^−1^, respectively (an endothermic reaction). Also, the computed free energy (*ΔG°* = 14.99 kcal mol^−1^) indicates a nonspontaneous process in atmospheric conditions. On the other hand, an NBO analysis reveals that thiophene oxidation via sulfur atom is difficult due to localizing the sulfur electrons on the π* orbitals. This subject will be discussed briefly in the NBO analysis section (see “Natural bond orbital (NBO) analysis”).

From a synthetic point of view, the addition of oxygenated compounds to thiophene and the production of thiophene-1-oxide or thiophene-S-oxide is an interesting subject for the synthesis of different derivatives of thiophene^57^. But, in the experiment, these reactions take place under special conditions, such as utilizing strong oxidative. Also, in a computational study, thiophene oxidation through sulfur atom by NO_3_ radical was unsuccessful^23^.

##### Oxygen addition to β-C

For the addition of HO_2_ to the β-C, two different paths are discussed. Two intermediates 1 and 4 are made by TS1 and TS4, respectively. The transition state TS1 involves the formation of a covalent bond due to the strong interaction (*ρ*(*r*_*LCP*_) = 0.1062 e bohr^−3^ and *∇*^2^*ρ*(*r*_*LCP*_) = 0.1280 e bohr^−5^) between the terminal oxygen of HO_2_ and β carbon. The computed relative energy (and energy barrier) for TS1 is 14.69 (21.33) kcal mol^−1^. In TS1, the critical distance between the atoms C3 and O10 is 1.81 Å. The similar C···O distance in TS4 (1.51 Å) is shorter than TS1, and it is 54.22 kcal mol^−1^ above the initial reactants.

In TS4, first, the oxygen atom (connected to a hydrogen atom) of HO_2_ approaches the β-C and constitutes a covalent bond (*ρ*(*r*_*LCP*_) = 0.2059 e bohr^−3^ and *∇*^2^*ρ*(*r*_*LCP*_) = − 0.3086 e bohr^−5^) with that carbon after that the hydrogen atom shifts to terminal oxygen. In comparison with TS1 and from the kinetic point of view, TS4 is an unfavorable saddle point due to having a high energy barrier. Complexes 1 and 4 are enantiomers of each other. The prior has R orientation and the last is S. The difference in energy between the enantiomers is 0.17 kcal mol^−1^, which the R enantiomer is more stable.

Finally, the kinetic and thermodynamic results obtained by three methods, B3LYP, CCSD(T), and BD(T), lead us to conclude that the attack of terminal oxygen to the α-C is more favorable than the β-C. Similar trends were also found in other thiophene addition reactions (NO_3_ by oxygen head, OH, and O_2_)^23,26,27^.

In the following section, we will see how 1 rearranges to other stable compounds through TS5 and TS6. And also, by starting from complex 4, it will be discussed how intermediate 7 is produced.

##### Rearrangement pathways of 1

The intermediate 1 can rearrange to 6 through TS6. This saddle point involves oxygen–oxygen bond breaking (*ρ*(*r*_*LCP*_) = 0.0461 e bohr^−3^ and *∇*^2^*ρ*(*r*_*LCP*_) = 0.2100 e bohr^−5^) and oxygen-carbon bond forming. Complex 6 includes a newly formed covalent bond (*ρ*(*r*_*LCP*_) = 0.2293 e bohr^−3^ and *∇*^2^*ρ*(*r*_*LCP*_) = − 0.2662 e bohr^−5^) between the oxygen O10 and the carbon C2, and a six-membered ring with one hydrogen bond (*ρ*(*r*_*LCP*_) = 0.0101 e bohr^−3^ and *∇*^2^*ρ*(*r*_*LCP*_) = 0.3289 e bohr^−5^) and a weak covalent interaction between the oxirane-like bicyclic product and hydroxyl radial. The AIM parameters of the six-membered ring are *ρ*(*r*_*rcp*_) = 0.0096 e bohr^−3^ and *∇*^2^*ρ*(*r*_*rcp*_) = 0.03778 e bohr^−5^. In the thiophene 3,4-epoxide, the distance of O10 with C2 and C3 are 1.47 Å and 1.47 Å, respectively. Also, The epoxy ring among C2, O10, and C3 atoms is confirmed by AIM as *ρ*(*r*_*rcp*_) = 0.1978 e bohr^−3^ and *∇*^2^*ρ*(*r*_*rcp*_) = 0.3542 e bohr^−5^. The continuation of the reaction from 6 happens by two routes. The first leads to form the OH and SC_2_H_2_OC_2_H_2_ (1,4-oxathiine) without entrancing any barrier. In 1,4-oxathiane, the oxygen atom is inserted into the C–C bond after cleavage of the interactions of six-membered rings. In another route, the hydroxyl radical has an electrophilic reaction through a six-membered ring with the S atom of 1,4-oxathiane. Thus, complex 15 is formed via TS15. 15 rearranges to 15-1 without passing through any TS. So, the distance between S and OH increases to 1.84 Å, and after that H7 atom shifts to O10 by surmounting TS16, resulting in thiophene-3-ol formation. Also, the hydroxyl radical of the previous step remains unchanged. So, the products, thiophene-3-ol and OH, are obtained by dissociation of the weak covalent bond between the 11O and 5S atoms (*ρ*(*r*_*LCP*_) = 0.0397 e bohr^−3^ and *∇*^2^*ρ*(*r*_*LCP*_) = 0.1184 e bohr^−5^) in 16. This process is exothermic (*ΔH°* = − 32.73 kcal mol^−1^) and spontaneous (*ΔG°* = − 31.29 kcal mol^−1^).

The rearrangement of 1 leads to thiophene 2,3-epoxide (another oxirane-like bicyclic product) and OH. The saddle point for this reaction is TS5 with relative energy of 18.36 kcal mol^−1^. In this transition state, a covalent bond (O–O) is cleavage (*ρ*(*r*_*LCP*_) = 0.0895 e bohr^−3^ and *∇*^2^*ρ*(*r*_*LCP*_) = 0.3303 e bohr^−5^) and a covalent bond (C1-O10) is forming simultaneously. In TS5, the bond lengths of C2-O10 and C1-O10 are 1.39 Å and 1.98 Å, respectively. In adduct 5, the distance of O10 from α and β carbons (the lengths of two sides) are 1.42 Å and 1.47 Å, respectively. The existence of an epoxy structure (three-membered ring) among C1, O10, and C2 atoms is also confirmed by an AIM analysis (*ρ*(*r*_*rcp*_) = 0.2017 e bohr^−3^ and *∇*^2^*ρ*(*r*_*rcp*_) = 0.3349 e bohr^−5^). The key intermediate 5 lays 12.57 kcal mol^−1^ below the initial reactants. In the exit channel, the final products can obtain by getting 7.07 kcal mol^−1^ and breaking the O10-H12 hydrogen bond (*ρ*(*r*_*LCP*_) = 0.0246 e bohr^−3^ and *∇*^2^*ρ*(*r*_*LCP*_) = 0.0918 e bohr^−5^) of complex 5. The standard enthalpy of this reaction is 5.98 kcal mol^−1^ indicating an endothermic reaction from the thermodynamic point of view. Also, Song et al.^27^ reported the same oxirane-like bicyclic product in the thiophene plus molecular oxygen reaction. They calculated relative energy for oxirane-like bicyclic product and ^3^O at the G4MP2 method was 52.45 kcal mol^−1^ above the initial reactants. Also, our calculated geometry for the oxirane-like bicyclic product is very similar to the structure suggested by Song et al. In the next section, we will investigate the reactions of the intermediate complex 5 by five different channels.

##### Rearrangement pathways of 5

In the first channel, the covalent bond between O10 and C1 atoms in 5 is ruptured (*ρ*(*r*_*LCP*_) = 0. 2527 e bohr^−3^ and *∇*^2^*ρ*(*r*_*LCP*_) = − 0.5355 e bohr^−5^), and a hydrogen atom (H8) is migrated simultaneously from C3 to C4, converting 5 into 9 through TS9. This intermolecular migration is called the NIH shift^58^. This mechanism is similar to the proposed one for thiophene-2-one synthesis in an experimental study^59^. In this process, the energy barrier is 42.04 kcal mol^−1^. The post-reactive complex 9 has a five-membered ring-like structure due to the formation of two van der Waals interactions. It is demonstrated that the electronic charge density of that ring is *ρ*(*r*_*rcp*_) = 0.0088 e bohr^−3^, and the Laplacian of density at ring critical point is *∇*^2^*ρ*(*r*_*rcp*_) = 0.0427 e bohr^−5^. The reaction can progress by 9 via two pathways. At the first route, 9 through an endergonic process and without entrancing any energy barrier converts directly to the 2H-3-thiophenone and OH products. But, the overall reaction R → 2H-3-thiophenone + OH is an exergonic reaction in the standard condition (*ΔH°* = − 37.16 kcal mol^−1^ and *ΔG°* = − 35.55 kcal mol^−1^). In the second path, the hydroxyl radial gets one hydrogen atom (H8) from 9. Then, a complex product (11) is formed with − 75.29 kcal mol^−1^ in relative energy. Some of this large stability is related to a six-membered ring structure in 11 due to the formation of two hydrogen bonds between thiophene-3-oxide and H_2_O. Also, the formation of this ring is proved by an AIM analysis (*ρ*(*r*_*rcp*_) = 0.0069 e bohr^−3^ and *∇*^2^*ρ*(*r*_*rcp*_) = 0.0350 e bohr^−5^). The thiophene-3-oxide and H_2_O products are produced directly from complex 11. These adducts are very stable and lay 66.53 kcal mol^−1^ under the initial reactants. Also, the sum of the standard Gibbs free energies of these products (*ΔG°* = − 66.24 kcal mol^−1^) shows that the transformation of the reactants to the mentioned adducts is a spontaneous reaction.

By TS10, the covalent bond (*ρ*(*r*_*LCP*_) = 0.2527 e bohr^−3^ and *∇*^2^*ρ*(*r*_*LCP*_) = − 0.5355 e bohr^−5^) between α and β carbons in 5 is broken and elongates to 1.74 Å, and the insertion of oxygen between two carbons happens (*r*_*α-C-O10*_ = 1.35 Å and *r*_*β-C-O10*_ = 1.45 Å). On the other hand, C2 reacts with hydroxyl radical and adduct 10 (6H-1, 3-oxathiin-6-ol) is produced with − 45.67 kcal mol^−1^. This saddle point has a six-membered ring ((*ρ*(*r*_*rcp*_) = 0.0311 e bohr^−3^ and *∇*^2^*ρ*(*r*_*rcp*_) = 0.1778 e bohr^−5^)). The energy height for TS10 is 25.01 kcal mol^−1^. From a thermodynamic point of view, the reaction is exothermic and spontaneous (*ΔG°* = − 35.55 kcal mol^−1^) in standard conditions.

In another pathway, complex 5 rearranges to the stable complex 8 (− 17.28 kcal mol^−1^) after passing TS8 with an energy barrier of 23.53 kcal mol^−1^. Also, the newly formed complex is converted to OH and a linear product, SC_4_H_4_O, by elimination of the hydrogen bond (*ρ*(*r*_*LCP*_) = 0.0270 e bohr^−3^ and *∇*^2^*ρ*(*r*_*LCP*_) = 0.0946 e bohr^−5^), between O12 and H11 through a spontaneous process (*ΔG°* = − 12.19 kcal mol^−1^). Besides, as mentioned above, these products can attain from 2. Cabanas et al.^22^ proposed experimentally a mechanism for the NO_3_ and thiophene reaction at room temperature. The mentioned linear adduct SC_4_H_4_O was also observed from the thiophene2,3-epoxide. However, in theoretical studies on the thiophene plus NO_3_ reaction, some linear sulfide compounds were reported as the final products^23,60^.

Isomerization of 8 to 12 and 13 happens by surmounting TS12 and TS13, respectively. These reactions require energy barriers of 47.13 and 28.31 kcal mol^−1^, respectively. The saddle points TS12 and TS13 involve H-shift from α-C to O10 and β-C, respectively. The H-shift in TS13 is the NIH shift. The hydrogen-bonded (*ρ*(*r*_*LCP*_) = 0.0230 e bohr^−3^ and *∇*^2^*ρ*(*r*_*LCP*_) = 0.0874 e bohr^−5^) post-reactive complex (12) is − 36.03 kcal mol^−1^ more stable than the original reactants. In the final step, 12 transforms directly to thiophene-2-ol and hydroxyl radical via breaking the O10–H12 bond. The thermodynamic parameters of the mentioned products are *ΔH°* = − 31.18 kcal mol^−1^ and *ΔG°* = − 29.64 kcal mol^−1^.

The post-reactive complex 13 can be converted to the corresponding products (13) by breaking the hydrogen bond between O10 and H12 (*ρ*(*r*_*LCP*_) = 0.0257 e bohr^−3^ and *∇*^2^*ρ*(*r*_*LCP*_) = 0.0923 e bohr^−5^), or can be rearranged to another isomer (14). The final products 13, 3H-2-thiophenone, and hydroxyl radical, are produced via an exothermic (− 38.98 kcal mol^−1^ in enthalpy) and spontaneous process (− 37.43 kcal mol^−1^ in Gibbs free energy).

Isomerization of 13–14 takes place by entrancing only 6.34 kcal mol^−1^ through TS14 in which the hydroxyl radical abstracts the H3 atom. The complex 14 contains a six-membered ring-like structure (*ρ*(*r*_*rcp*_) = 0.0058 e bohr^−3^ and *∇*^2^*ρ*(*r*_*rcp*_) = 0.0292 e bohr^−5^), including two hydrogen bond interactions. The final products are produced directly by breaking the ring structure in 14 without any barrier. The thiophene-2-oxide and H_2_O are the final products. They are also the most stable and favorable ones among all products thermodynamically. In the standard condition, this process is spontaneous (*ΔG°* = − 75.51 kcal mol^−1^) and exothermic (*ΔH°* = − 76.39 kcal mol^−1^).

##### Rearrangement pathways of 4

The intermediate 4 can rearrange to 7 through the saddle point TS7. In TS7, the hydroxyl radical reacts with C4 and the bond between C4 and S elongates to 1.92 Å in 7. Complex 7 lays 40.94 kcal mol^−1^ below the reactants. There is a six-membered ring in 7 (*ρ*(*r*_*rcp*_) = 0.0231 e bohr^−3^and *∇*^2^*ρ*(*r*_*rcp*_) = 0.1260 e bohr^−5^) containing the O10-H12 hydrogen bond (*ρ*(*r*_*LCP*_) = 0.0245 e bohr^−3^ and *∇*^2^*ρ*(*r*_*LCP*_) = 0.0937 e bohr^−5^). The complex 7 by an intramolecular hydrogen shift transforms to a stable product 17. In the saddle point TS17, the atom H8 on C3 is abstracted by O10, and hydroxyl radical is formed, and also the distance between C4 and S decreases to 1.86 Å. Then, 17 (2,5-dihydrothiophene-2,3-diol) is produced with relative energy of − 65.01 kcal mol^−1^. This process is spontaneous (*ΔG°* = − 50.96 kcal mol^−1^) and exothermic (*ΔH°* = − 62.64 kcal mol^−1^).

### Natural bond orbital (NBO) analysis

NBO analysis is a suitable quantum chemistry method for understanding inter-and intra-molecular interactions, and also is used particularly for charge transfer interpretation. In Lewis structures, a deviation from an ideal form is observed when a transfer of occupancy occurs from the localized NBOs of the idealized Lewis structure into the unfilled non-Lewis orbitals due to delocalization of electron density among bonding and lone pair orbitals (as occupied Lewis-type NBOs), and nonbonding and Rydberg orbitals (as unoccupied non-Lewis type NBOs). So, a correction denoted as delocalization correction through a stabilizing donor–acceptor interaction is required to the zero-order Lewis structures^61^. The energies of the second-order perturbation theory can predict the energies of such interactions. The second-order stabilization energy E_2_ between a donor NBO (i) and an acceptor NBO (j) regarding the respective delocalization can be assessed as:

1 $$E_{2} = \Delta E = q_{i} \left[ {\frac{{F_{{i,j}}^{2} }}{{\varepsilon _{i} - \varepsilon _{j} }}} \right],$$

where q_i_ is the orbital occupancy, *ε*_*i*_ and *ε*_*j*_ are diagonal elements of the NBO Fock matrix, and *F*_*i,j*_ is the off-diagonal elements^62^. The second-order perturbation theory analysis of the Fock matrix by using NBO calculations for some species is represented in Table 3.

**Table 3:** The second-order perturbation theory analysis of the Fock matrix using NBO calculations for some species in the thiophene plus hydroperoxyl radical reaction.

Compound	Donor NBOs	Occupancy (e)	Acceptor NBOs	occupancy(e)	E_2_ (kcal mol^−1^)
Thiophene	n_S(1)_	1.8033	π*(C_1_–C_2_)	0.29876	21.6
			π*(C_3_–C_4_)	0.0145	21.6
C_4_H_4_S…HO_2_	n_S(2)_	1.6091	π*(C_1_–C_2_)	0.2891	21.76
			π*(C_3_–C_4_)	0.0148	22.57
TS1	n_S(2)_	0.86584	π*(C_1_–C_2_)	0.12529	10.87
			π*(C_3_–C_4_)	0.21062	5.50
	n_O10(3)_	0.86702	π*(C_3_–C_4_)	0.21062	48.54
	π*(C_1_–C_2_)	0.07594	σ*(C_3_–O_10_)	0.05984	16.66
TS2	n_S(2)_	0.84425	π*(C_1_–C_2_)	0.20183	14.22
	n_S(2)_	0.81376	σ*(C_1_–O_12_)	0.08370	7.51
	π(C_1_–C_2_)	0.88902	π*(C_1_–C_2_)	0.18440	16.29
	n_O10(3)_	0.88922	π*(C_1_–C_2_)	0.20183	30.54
TS3	n_S(1)_	1.8932	π*(C_3_–C_4_)	0.2418	2.41
	n_O10(3)_	0.94199	σ*(S_5_–O_10_)	0.19990	10.35
	n_O11(3)_	0.72799	σ*(S_5_–O_10_)	0.19990	41.68
	σ*(S_5_–O_10_)	0.19990	π*(C_1_–C_2_)	0.16338	18.38
			π*(C_3_–C_4_)	0.17995	30.98
TS4	n_S(1)_	1.9858	π*(C_1_–C_2_)	0.1296	10.72
	σ(C_3_–O_10_)	0.91853	π*(C_3_–C_4_)	0.17169	79.62
	σ(C_3_–H_8_)	0.92072	σ*(C_3_–O_10_)	0.07194	42.63
	σ*(C_3_–O_10_)	0.07194	π*(C_3_–C_4_)	0.17169	414.88
	n_O12(3)_	1.8730	σ*(O_10_–H_11_)	0.1261	40.98

According to the data reported in Table 3, there are strong interactions between the lone pairs of the atom S and the unoccupied π*(C_1_–C_2_) and π*(C_3_–C_4_) orbitals. The mentioned stabilization energies are going from ~ 22.57 for pre-reactive complex (C_4_H_4_O…HO_2_) to less than ~ 2.41 for TS3. In comparison with thiophene, the n_S(2)_ $\to$ π*(C_1_–C_2_) stabilization energy (E_2_) strongly influences the activation energies of TS1(E_2_ =  ~ 10.87 kcal mol^−1^) and TS2 (E_2_ =  ~ 14.22 kcal mol^−1^). Therefore, charge-transfer delocalization makes TS2 more stable than TS1. In TS2, there is also another interaction between the lone pair electrons of sulfur (occupancy = 0.81376) and the unoccupied σ*(C_1_–O_12_) orbital (occupancy = 0.08370) with stabilization energy of 7.51 kcal mol^−1^. In TS3, the oxygen atom is added to sulfur then this stabilization energy is reduced to 2.41 kcal mol^−1^ (n_S(1)_ $\to$ π*(C_3_–C_4_)). The stabilization energy for n_S(1)_ $\to$ π*(C_1_–C_2_) is 10.72 kcal mol^−1^ for TS4. The TS4 according to the barrier height of 54.22 kcal mol^−1^ is an unfavorable pathway. The analyzed data by using the NBO approach for TS4 show that, on one hand, there is the interaction of σ (C_3_–H_8_) $\to$ σ*(C_3_–O_10_) with E_2_ =  ~ 42.83 kcal mol^−1^ and, on the other hand, another interaction exists as σ*(C_3_–O_10_) $\to$ π*(C_3_–C_4_) with significant energy of 414.88 kcal mol^−1^. These interactions are responsible for the unstability of this transition state. Also, the interaction of lone pair electrons of O12 with the anti-bonding orbitals σ*(O_10_–H_11_) (E_2_ =  ~ 40.98 kcal mol^−1^) leads to the H-abstraction by O12.

### Kinetic rate constant calculation

**Figure 5:**
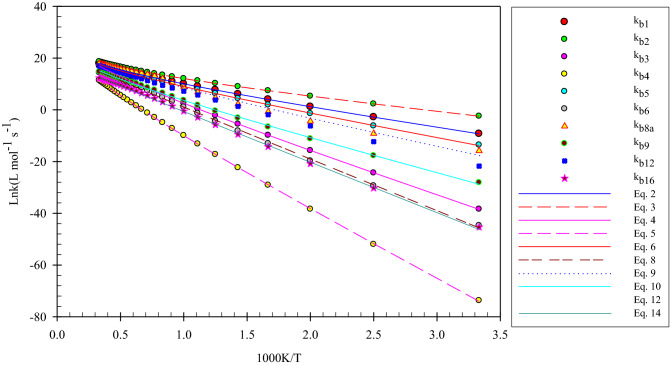
The Arrhenius plots of selected pathways calculated by TST and RRKM theories and predicted by equations 2–14.

The rate constant calculations are discussed in more detail in supporting information. The rate constants of all forward and reverse reactions for generations of all stationary points using the pathways calculated in the suggested multiwell-multichannel PES are listed in Supplementary Table S6 at different temperatures (300–3000 K). The Arrhenius plots are sketched in Fig. 5 for all minimum structures over a temperature range from 300 to 3000 K, and pressure of 1 bar using RRKM theory. These plots are used to show the influence of temperature on the production of the above-discussed intermediates and adducts.

#### Forward reactions

To predict the rate constants computed by mixing TST and RRKM theories, the non-Arrhenius forms of the results obtained at 300–3000 K are extracted by the non-linear least-squares fitting method. All the following rate expressions are bimolecular in the unit of L mol^−1^ s^-1^.

2 $$k_{{b1}} = 3.93 \times 10^{5} \left( {\frac{T}{{300}}} \right)^{{3.16 \pm 0.01}} \exp \left[ { - \frac{{(13.22 \pm 0.02)\,{\text{kcal}}\,{\text{mol}}^{{ - 1}} }}{{RT}}} \right],$$

3 $$k_{{b2}} = 4.03 \times 10^{5} \left( {\frac{T}{{300}}} \right)^{{3.14 \pm 0.01}} \exp \left[ { - \frac{{(9.18 \pm 0.01)\,{\text{kcal}}\,{\text{mol}}^{{ - 1}} }}{{RT}}} \right],$$

4 $$k_{{b3}} = 1.32 \times 10^{6} \left( {\frac{T}{{300}}} \right)^{{3.50 \pm 0.04}} \exp \left[ { - \frac{{(31.36 \pm 0.06)\,{\text{kcal}}\,{\text{mol}}^{{ - 1}} }}{{RT}}} \right],$$

5 $$k_{{b4}} = k_{{b7}} = k_{{b17}} = 3.00 \times 10^{4} \left( {\frac{T}{{300}}} \right)^{{4.17 \pm 0.11}} \exp \left[ { - \frac{{(50.29 \pm 0.19)\,{\text{kcal}}\,{\text{mol}}^{{ - 1}} }}{{RT}}} \right],$$

6 $$k_{{b5}} = 1.39 \times 10^{6} \left( {\frac{T}{{300}}} \right)^{{2.69 \pm 0.08}} \exp \left[ { - \frac{{(16.68 \pm 0.15)\,{\text{kcal}}\,{\text{mol}}^{{ - 1}} }}{{RT}}} \right],$$

7 $$k_{{b6}} = 5.93 \times 10^{8} \left( {\frac{T}{{300}}} \right)^{{0.50 \pm 0.41}} \exp \left[ { - \frac{{(39.26 \pm 0.73){\text{kcal}}\,{\text{mol}}^{{ - 1}} }}{{RT}}} \right],$$

8 $$k_{{b8}} = k_{{b13}} = k_{{b14}} = 1.40 \times 10^{6} \left( {\frac{T}{{300}}} \right)^{{2.68 \pm 0.08}} \exp \left[ { - \frac{{(16.69 \pm 0.15){\text{kcal}}\,{\text{mol}}^{{ - 1}} }}{{RT}}} \right],$$

9 $$k_{{b8a}} = 1.95 \times 10^{6} \left( {\frac{T}{{300}}} \right)^{{3.17 \pm 0.82}} \exp \left[ { - \frac{{(19.08 \pm 1.44){\text{kcal}}\,{\text{mol}}^{{ - 1}} }}{{RT}}} \right],$$

10 $$k_{{b9}} = k_{{b11}} = 3.07 \times 10^{5} \left( {\frac{T}{{300}}} \right)^{{2.85 \pm 0.31}} \exp \left[ { - \frac{{(24.69 \pm 0.55)\,{\text{kcal}}\,{\text{mol}}^{{ - 1}} }}{{RT}}} \right],$$

11 $$k_{{b10}} = 1.24 \times 10^{6} \left( {\frac{T}{{300}}} \right)^{{2.52 \pm 0.07}} \exp \left[ { - \frac{{(16.60 \pm 0.12)\,{\text{kcal}}\,{\text{mol}}^{{ - 1}} }}{{RT}}} \right],$$

12 $$k_{{b12}} = 8.69 \times 10^{6} \left( {\frac{T}{{300}}} \right)^{{2.16 \pm 0.17}} \exp \left[ { - \frac{{(22.76 \pm 0.30)\,{\text{kcal}}\,{\text{mol}}^{{ - 1}} }}{{RT}}} \right],$$

13 $$k_{{b15}} = 5.39 \times 10^{8} \left( {\frac{T}{{300}}} \right)^{{0.50 \pm 0.41}} \exp \left[ { - \frac{{(39.26 \pm 0.73){\text{kcal}}\,{\text{mol}}^{{ - 1}} }}{{RT}}} \right],$$

14 $$k_{{b16}} = 9.71 \times 10^{7} \left( {\frac{T}{{300}}} \right)^{{0.35 \pm 0.37}} \exp \left[ { - \frac{{(38.47 \pm 0.65){\text{kcal}}\,{\text{mol}}^{{ - 1}} }}{{RT}}} \right].$$

All of the Arrhenius rate expressions reveal that the pre-exponential factors have a temperature dependency. As mentioned above, these expressions are the results of fitting, so they have no physical meaning. Our computed high-pressure limit rate constants for addition reactions include the addition to β-C, k_b1_ (and k_b4_), the addition to α-C, k_b2_, and the addition to S atom, k_b3_. They have the rate expressions of $3.93 \times 10^{5} \left( {\frac{T}{{300}}} \right)^{{3.16}} \exp \left( { - \frac{{13.22\,{\text{kcal}}\,{\text{mol}}^{{ - 1}} }}{{RT}}} \right)$ (and $4.03 \times 10^{5} \left( {\frac{T}{{300}}} \right)^{{3.14}} \exp \left( { - \frac{{9.18\,{\text{kcal}}\,{\text{mol}}^{{ - 1}} }}{{RT}}} \right)$), $1.32 \times 10^{6} \left( {\frac{T}{{300}}} \right)^{{3.50}} \exp \left( { - \frac{{31.36\,{\text{kcal}}\,{\text{mol}}^{{ - 1}} }}{{RT}}} \right)$, and $3.00 \times 10^{4} \left( {\frac{T}{{300}}} \right)^{{4.17}} \exp \left( { - \frac{{50.29\,{\text{kcal}}\,{\text{mol}}^{{ - 1}} }}{{RT}}} \right)$ L mol^−1^ s^−1^, respectively. These results demonstrated that addition to the carbon α-C is more favorable than β-C in the mentioned temperature range. The addition rate to α-C is 50.59, 3.86, and 2.81 times larger than that of β-C at 300, 1500, and 3000 K, respectively (see Supplementary Table S5). Also, the rate constants of the addition reaction of the S atom, k_3_, reveal that in small temperatures, sulfur atom does not react with hydroperoxyl radical, but at a temperature above 1000 K, this reaction has a good chance to occur. The small rate constant at low temperatures confirms that this reaction has a small contribution to the atmospheric degradation of thiophene. The calculated rate constant for the generation of 4 via the addition along with hydrogen transfer reaction, k_4_, is small due to the large energy barrier. This reaction has a relatively good contribution to thiophene degradation at temperatures above 2000 K. Also, this step is a rate-determining step for the generations of 7 and 17. Thus, these species have the same rate constant. For the mentioned rate-determining step, the calculated rate constants at 300, 1500, and 3000 K are 1.07E−33, 2.33E+01, and 5.42E+05 s^−1^, respectively (see Supplementary Table S6). For 5, the obtained rate expression in the studied temperature range is $1.39 \times 10^{6} \left( {\frac{T}{{300}}} \right)^{{2.69}} \exp \left( { - \frac{{16.68\,{\text{kcal}}\,{\text{mol}}^{{ - 1}} }}{{RT}}} \right)$ L mol^−1^ s^−1^, indicating a two-step reaction with a good contribution to thiophene elimination in the low temperatures. The high-pressure limit rate constant for 8 and 10 is similar to 5, demonstrating the possibility of a third step reaction, even at low temperatures (see Supplementary Table S5). The rate-determining step of these reactions has the rates of 1.03E+07, 1.14E+05, and 1.46E+07 s^−1^ at 300, 1500, and 3000 K, respectively. The oxirane-like bicyclic species, 6, has a non-Arrhenius expression in the high-pressure limit as $5.93 \times 10^{8} \left( {\frac{T}{{300}}} \right)^{{0.50}} \exp \left( { - \frac{{39.26\,{\text{kcal}}\,{\text{mol}}^{{ - 1}} }}{{RT}}} \right)$ L mol^−1^ s^−1^. Then, the production of this complex takes place above 1000 K in atmospheric conditions. Since the rate-determining step for 15 is the same as 6, they have a similar rate for generation, but 16 due to having large barrier energy in the final step has a smaller rate at the same temperatures ($9.71 \times 10^{7} \left( {\frac{T}{{300}}} \right)^{{0.35}} \exp \left( { - \frac{{38.47\,{\text{kcal}}\,{\text{mol}}^{{ - 1}} }}{{RT}}} \right)$ L mol^−1^ s^−1^). According to Supplementary Table S6, the rate constant of the rate-determining step of 6 and 15 is 3.03E−07, 5.23E+02, and 2.27E+05 s^−1^ at 300, 1500, and 3000 K, respectively, and for 16 they are 4.29E−19, 4.54E−02, and 1.11E+01 s^−1^, respectively, at the same temperatures. About complex 9, its generation pathway contains three saddle points and the final step has a large barrier height. This complex is produced above a temperature of 900 K with a rate expression of $3.07 \times 10^{5} \left( {\frac{T}{{300}}} \right)^{{2.85}} \exp \left( { - \frac{{24.69\,{\text{kcal}}\,{\text{mol}}^{{ - 1}} }}{{RT}}} \right)$ L mol^−1^ s^−1^. The adduct 11 is created after passing TS11 from complex 9, but the final step in the generation pathway of 11 has a small barrier height. So, 9 and 11 have a similar rate. Produced by a four-step pathway, the adduct 12 has a good contribution above 700 K with a rate expression of $8.69 \times 10^{6} \left( {\frac{T}{{300}}} \right)^{{2.16}} \exp \left( { - \frac{{22.76\,{\text{kcal}}\,{\text{mol}}^{{ - 1}} }}{{RT}}} \right)$ L mol^−1^ s^−1^. Another species that is produced through a four-step reaction is 13. It is generated above 600 K. Also, product 14 has a rate similar to 13.

#### Reverse reactions

As shown in Table S6, the rate constants of unimolecular reactions of 1, 2, 3, and 4 for conversion to the C_4_H_4_S…HO_2_ complex (reverse reactions) are more than the unimolecular respective forward reactions at low temperatures. Our computed the unimolecular rate constants for back reactions of the above discussed addition reactions (1, 2, 3, and 4 to C_4_H_4_S…HO_2_) are 7.86E+08, 1.19E+07, 4.05E−06, and 9.04E−03 s^−1^ at 300 K, respectively. This does not mean that after the production of these species, they rapidly go back to the pre-reactive complex or reactants. As is well known, many atmospheric reactions occur under the radiation of sunlight. So, the reaction energy barrier supply by radiation. Also, as we know, radiation has broad wavelengths. When radiation with high energy impact an atmospheric species, a part of its energy is consumed for activation energy, and other parts are consumed for conversion of the post-reactive complex to corresponding products and or may supply the next steps barrier energies. The reverse reactions happen when radiation has just the energy around activation energy. And so, due to the unstability of the newly formed species along with small reverse barrier energy, it comes back to the initial reactants in the presence of weak radiations.

#### Altitude and reactivity

**Figure 6:**
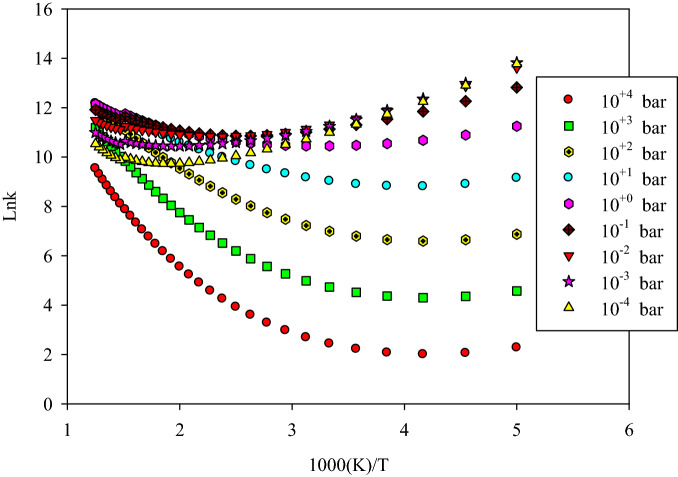
The pressure dependent rate constant calculated by RRKM theory for the reaction of HO_2_ addition to α carbon.

**Figure 7:**
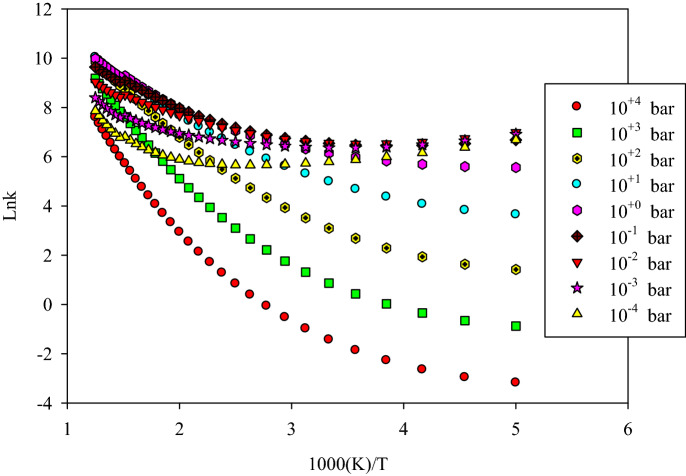
The pressure dependent rate constant calculated by RRKM theory for the reaction of HO_2_ addition to β carbon.

It is well known that the reactivity of some atmospheric species can be affected by altitude in different areas of the atmosphere. This is seen mainly in the troposphere (the altitude from 0 to 12 km) where the temperature varies 6.49 K for the 1 km change in height. It can be concluded that the variation in altitude is a function of both temperature and pressure. These factors can vary the thermochemical properties, kinetics, and so reactivity of some atmospheric reactions. The explicit effect of temperature on the reaction was discussed in the previous section. Herein, the influence of pressure is considered only on the rate constants of the above-discussed addition reactions by the strong collision approach. The nitrogen of the atmosphere is selected as the colliding (bath) gas due to its high concentration. The pressure dependent rate constant, *k*(*T*,*p*), for the addition of HO_2_ to α and β carbons, are tabulated in Table S7 and shown in Figs. 6 and 7, respectively. Also, the addition to the S atom is sketched in Supplementary Fig. S1.

The significant results about the effect of pressure on the pathways of the α and β addition reactions are observed by increasing pressure in the 200–800 K temperature range. It is remembered that the pressure dependent rate constant can be written as^63,64^:

15 $$k(T,p) = \frac{{k_{\infty } }}{{1 + \frac{{k_{\infty } }}{{k_{0} }}}}.$$

This equation indicates that the ratio of *k*_*∞*_/*k*_0_ is an important term in the computation of the fall of regime rate constants. Through this ratio, the effect of pressure can be represented clearly on any gas phase reaction occurring in atmospheric conditions. The ratio *k*_*∞*_/*k*_0_ at 300, 400, and 500 K, is 1.92E−04, 2.18E−03, and 1.51E−02, respectively, for α addition and is 7.29E−04, 8.01E−03, and 5.24E−02, respectively, for β addition. These results show that this ratio increases under the considered temperature range. Therefore, *k*(*T*,*p*) is decreased.

On the other hand, reducing the obtained rate constants at any temperature into the respective rate constants at 1 bar makes a better insight into the effect of pressure on the rate of reaction at the same temperature. The reduced values for α addition at 300 K, *k*_2_(300 K, *p*)/*k*_2_(300 K,1 bar), in *p* = 10^–2^, 10^–1^, 1, 10, and 10^2^ bar are 2.35, 1.98, 1, 2.40E−01, and 3.15E−02, respectively. The corresponding values for β addition, *k*_1_(300 K,* p*)/*k*_1_(300 K,1 bar), are 1.50, 1.49, 1, 3.17E−01, and 4.74E−02, respectively. These results also confirm that the addition rate constants decrease with increasing pressure over the studying temperature range.

In addition, the pressure has no sensible influence on the α and β addition reactions at temperatures above 800 K. This may relate to the reaction cross-section. The reaction cross-section for the addition reactions increases by temperature because the corresponding rate constants increase by temperature. In high temperatures, the reaction cross-section is high, which creates facilitated conditions for the happening reaction. Thus, the effect of pressure is negligible. In general, it can be concluded that the thiophene degradation by the addition reactions takes place at low pressures and high temperatures. Similar behavior is expected for the title reaction proceeding through the suggested multiwell-multichannel PES at low pressures and high temperatures.

The same calculations for S and β (by TS4) addition reactions show that the effect of pressure on those reactions is not substantial and the change in the values of rate constants is small (see Supplementary Figs. S1 and S2 and Supplementary Table S7). Finally, the findings of this section can be used as a model to interpret the reactivity of other aromatic compounds with HO_2_ at different altitudes.

## Conclusion

This work presented mechanistic and dynamic data for all possible consumption pathways of thiophene molecule and unimolecular degradation of its products under combustion conditions through a multiwell-multichannel reaction. Hydroperoxyl radical plays a key role in the generation of tropospheric ozone, but our results showed that it converts mainly to OH and H_2_O in the same conditions. The modified Arrhenius rate expressions were provided for combustion modeling of title reaction to account for atmospheric oxidation of thiophene. For the suggested multiwell-multichannel reaction, the potential energy surface was determined at the B3LYP, CCSD(T), and BD(T) methods. The transition state theory along with the chemical master equation technique (by means of the RRKM theory) was employed to investigate the dynamics and kinetics of all reaction pathways in the considered multiwell-multichannel reaction over a broad temperature range of 300–3000 K and pressure range of 1E−04 to 1E+04 bar. For computing treatment of title reaction at the high-pressure limit, TST theory was used to calculate the rate constants of addition reactions. And for investigating the behavior of it at 1 atm, the rate constants of all channels (unimolecular reactions) were calculated by the RRKM theory. So, the total rate and internal unimolecular conversion rate of each intermediate and final product from initial reactants were computed. Also, this theory was used to evaluate the rate constants of the HO_2_ addition to α and β carbons in the falloff regime. The negative pressure dependent rate constants were observed with decreasing altitude. According to the calculated rate constants, the addition to α and β carbons were the more favorable pathways than the S site at low temperatures and pressures. However, addition to S site and multi-step reactions could play a pivotal role in the atmospheric conditions by temperature increasing. Rearrangements of complex 5 to important adducts by the considered paths confirmed that it is a key complex. The strong interactions among the lone pairs electrons of the sulfur atom and unoccupied π* orbitals of thiophene were established by the NBO analysis, leading to a decrease in the activation energy of some transition states. The computed standard enthalpies and Gibbs free energies were negative for the many products obtained in this study. Also, the results revealed that thiophene-2-oxide is the main thermodynamic product (*ΔG°* = − 75.51 kcal mol^−1^ and *ΔH°* = − 76.39 kcal mol^−1^). As a reaction of cyclic compounds, the insertion of an oxygen atom to the aromatic ring (the oxidation process via π bonds) was also observed, and two oxathiine adducts were yielded.

## Supplementary Information

Supplementary Information.
