# Supplementary material for: Chemical insights into the atmospheric oxidation of thiophene by hydroperoxyl radical
Source: Sci Rep. 2021 Jun 22;11:13049. doi: 10.1038/s41598-021-92221-z (PMC8219665; doi:10.1038/s41598-021-92221-z)
Supplement: Supplementary file 1 — Supplementary Information. [file 41598_2021_92221_MOESM1_ESM.docx]

**Chemical insights into the atmospheric oxidation of thiophene by hydroperoxyl radical**

Maryam Seyed Sharifi^*^, Hamed Douroudgari^*^, Morteza Vahedpour

Department of Chemistry, University of Zanjan, PO Box 38791-45371, Zanjan, Iran

E-mail: m.ssharifi@znu.ac.ir

E-mail:  [douroudgari@znu.ac.ir](mailto:%20douroudgari@znu.ac.ir)

**Details of rate constant calculations**

Eyring transition state theory (TST) based on statistical mechanics is a familiar theory for calculating the rate constants of chemical reactions at high-pressure limit [^1^](#_ENREF_1). For a TST calculation, an accurate transition structure and a precise energy barrier are needed. As shown in scheme 1, the addition reactions between thiophene and HO_2_ happen through a two-step reaction mechanism [^2^](#_ENREF_2). The first step includes the formation of a rapid equilibrium between the isolated reactants and the van der Waals pre-reaction collision complex C_4_H_4_S…HO_2_. Thus,

(2)

where *k_f_* and *k_r_* denote the forward and reverse rate constants. And the second step is a conversion of the formed complex through different a saddle point to the related products:

(3)

where *k_uni_,_conv_* is the unimolecular rate coefficient of conversion of the formed complex to products. The equilibrium constant of the first step is calculated by

$\frac{k_{f}}{k_{r}}=K_{eq}=\frac{Q_{comp}}{Q_{C_{4}H_{4}S}Q_{HO_{2}}}\exp(-\frac{E_{comp}-E_{R}}{k_{B}T})$ (4)

where *Q_i_* and *E_i_* are the partition function and energy of i species, respectively. *R* and *comp* refer to reactants and associated complex, respectively. Also, the unimolecular rate coefficient for the second step is computed by TST theory as

$k_{uni}=\sigma\frac{k_{B}T}{h}\frac{Q_{TS}}{Q_{comp}}\exp(-\frac{E_{TS}-E_{comp}}{k_{B}T})$ (5)

where σ is the reaction degeneracy. *k_B_*, *h*, *T* and are Boltzmann’s constant, Planck’s constant, and temperature, respectively. *TS* denotes transition state. Finally, the following equation is used for calculating the rate constant of bimolecular reactions.

$k_{b}=K_{eq}k_{uni}$ (6)

For inclusion of the tunneling effect, a correction is added to equation 6. So, we have

$k_{b}=\kappa K_{eq}k_{uni}$ (7)

The tunneling correction is defined as the ratio of the quantum mechanical barrier crossing rate to the corresponding classical mechanical one. The Eckart potential function by considering an unsymmetrical barrier is widely used to estimate quantum mechanical tunneling corrections to calculate the accurate rate constants of reaction channels theoretically [^3^](#_ENREF_3). It is defined through the following integral over the threshold energy E as

$\kappa=\exp(\frac{E_{b}}{k_{B}T})\int_{E_{0}}^{\infty} P^{T}(E)\times\exp(-\frac{E}{k_{B}T})\frac{dE}{k_{B}T}$ (8)

where *E_b_* is the barrier height of the calculated path, and *P^T^(E)* is the tunneling transmission probability that depends on three parameters.

$P^{T}(E)=\frac{\cosh(a+b)-\cosh(a-b)}{\cosh(a+b)+\cosh(c)}$ (9)

in which

$a=\frac{2\pi\sqrt{2\mu E}}{h\alpha}$ (10)

$b=\frac{2\pi\sqrt{2\mu(E-\Delta E)}}{h\alpha}$ (11)

(12)

where *ΔE* is the reaction energy that defines the difference of energy between the sum of products and the sum of reactants. Other parameters have been defined elsewhere[^4^](#_ENREF_4).

The rate constant for unimolecular reactions is calculated by the RRKM theory [^5^](#_ENREF_5),[^6^](#_ENREF_6). In RRKM theory, first, the energy-dependent rate constant, *k(E)*, is calculated through

$k(E)=\frac{\sigma N^{\neq}(E-E_{0})}{h\rho(E)}$ (13)

where $\sigma$ is the reaction degeneracy, $N^{\neq}(E-E_{0})$is the sum states of transition state from *0* to$(E-E_{0})$, $\rho(E)$denotes the density of state of reactants. Finally, the thermal rate constant *k(T)* in the canonical form is computed using a regular equation as follows

$k(T)=\frac{1}{Q(T)}\int k(E)\rho(E)\exp(-\beta E)dE$ (14)

where *Q(T)* represents the partition function of reactants and $\beta=\frac{1}{k_{B}T}$. As we know, the Lennard-Jones parameters necessary for RRKM calculations. The mentioned parameters are not reported experimentally for thiophene. Thus, we used Tee[^7^](#_ENREF_7) relations ($\frac{\sigma}{\text{Å}}=2.3647\times(\frac{T_{c}}{P_{c}})^{(1/3)}$ and $\frac{\varepsilon}{\text{k}}=0.7740\times T_{c}$) to estimate the Lennard-Jones parameters of thiophene. The obtained results are *σ* *=* 5.180 Å and *ε/k_B_* *=* 448.460 K. For HO_2_, the reported values are *σ* *=* 3.458 Å and *ε/k_B_* *=* 107.400 K. Also, atmospheric nitrogen due to having a large concentration is selected as colliding gas with *σ* *=* 3.621 Å and *ε/k_B_* *=* 97.530 K [^8^](#_ENREF_8). As standard software for calculating the rate constants of all pathways, the Gpop program for bimolecular reactions and Ssumes program for unimolecular reactions are used [^9^](#_ENREF_9),[^10^](#_ENREF_10).

**Figure S1.** The pressure dependent rate constant calculated by RRKM theory for the reaction of HO_2_ addition to S atom.

**Figure S2.** The pressure dependent rate constant calculated by RRKM theory for the reaction of HO_2_ addition to β carbon by TS4.

| Species | $\left\langle S^{2}\right\rangle{}^{a}$ | $\left\langle S^{2}\right\rangle^{b}$ |
| --- | --- | --- |
| HO_2_ | 0.7539 | 0.7500 |
| CR | 0.7538 | 0.7500 |
| 1 | 0.7564 | 0.7500 |
| 2 | 0.7708 | 0.7501 |
| 3 | 0.7542 | 0.7500 |
| 4 | 0.7569 | 0.7500 |
| 5 | 0.7523 | 0.7500 |
| 6 | 0.7717 | 0.7502 |
| 7 | 0.7553 | 0.7500 |
| 8 | 0.7522 | 0.7500 |
| 8a | 0.7535 | 0.7500 |
| 9 | 0.7543 | 0.7500 |
| 10 | 0.7556 | 0.7500 |
| 11 | 0.7633 | 0.7501 |
| 12 | 0.7522 | 0.7500 |
| 13 | 0.7522 | 0.7500 |
| 14 | 0.7677 | 0.7502 |
| 15 | 0.7569 | 0.7500 |
| 15-1 | 0.7649 | 0.7500 |
| 16 | 0.7588 | 0.7500 |
| 17 | 0.7714 | 0.7501 |
| thiophene-3-oxide | 0.7664 | 0.7501 |
| thiophene-2-oxide | 0.7687 | 0.7502 |
| OH | 0.7522 | 0.7500 |
| TS1 | 0.7744 | 0.7502 |
| TS2 | 0.7834 | 0.7505 |
| TS3 | 0.7695 | 0.7501 |
| TS4 | 0.7593 | 0.7501 |
| TS5 | 0.8073 | 0.7504 |
| TS6 | 0.9657 | 0.7538 |
| TS7 | 0.7662 | 0.7502 |
| TS8 | 0.7782 | 0.7502 |
| TS8a | 0.7889 | 0.7503 |
| TS9 | 0.7743 | 0.7501 |
| TS10 | 0.7728 | 0.7501 |
| TS11 | 0.7579 | 0.7500 |
| TS12 | 0.7523 | 0.7500 |
| TS13 | 0.7523 | 0.7500 |
| TS14 | 0.7572 | 0.7500 |
| TS15 | 0.7686 | 0.7502 |
| TS16 | 0.8060 | 0.7505 |
| TS17 | 0.7692 | 0.7502 |

a) Before spin annihilation.

b) After spin annihilation.

**Table S1.** The spin expectation values $\left\langle S\right\rangle$ calculated at the B3LYP/6-311+g(d,p) level.

| Species | *ΔH°* | *ΔG°* | *ΔE°* | *TΔS°* |
| --- | --- | --- | --- | --- |
| Thiophene + HO_2_ | 0 | 0 | 0 | 0 |
| 1 | 16.14 | 6.83 | 13.33 | -10.93 |
| 4 | 16.22 | 6.81 | 13.38 | -10.84 |
| SCHOCHC_2_H_2_ + OH | 5.88 | 8.46 | 5.98 | -2.48 |
| 5 | 6.52 | -11.24 | 3.26 | -2.49 |
| 2 | 5.99 | 22.29 | 3.26 | -11.15 |
| SC_4_H_4_O + OH | -3.45 | -24.06 | -3.73 | 0.36 |
| 3 | 19.15 | 8.89 | 16.06 | -9.99 |
| C_4_H_4_SO + OH | 5.98 | 5.98 | 20.88 | -1.71 |
| 6 | 24.57 | 16.27 | 22.03 | -11.95 |
| SC_2_H_2_OC_2_H_2_ + OH | 1.51 | -17.08 | -2.08 | -1.66 |
| 7 | -31.14 | -39.24 | -33.40 | -12.15 |
| 8 | -6.17 | -19.96 | -4.37 | -6.46 |
| 8a | -7.97 | -21.59 | 20.54 | -6.63 |
| 9 | -34.01 | -45.14 | -37.42 | -9.12 |
| 2H-3-thiophenone + OH | -29.27 | -47.90 | -32.85 | -1.62 |
| 10 | -34.24 | -42.60 | -32.44 | -11.89 |
| 11 | -68.43 | -79.58 | -66.64 | -9.10 |
| thiophene-3-oxide + H_2_O | -62.70 | -81.98 | -64.63 | -0.97 |
| 12 | -25.78 | -38.33 | -29.32 | -7.70 |
| thiophene-2-ol + OH | -22.73 | -41.43 | -26.45 | -1.54 |
| 13 | -35.00 | -46.85 | -38.37 | -8.40 |
| 3H-2-thiophenone + OH | -30.38 | -49.08 | -34.01 | -1.55 |
| 14 | -77.96 | -89.68 | -81.57 | -8.53 |
| thiophene-2-oxide + H_2_O | -74.87 | -73.39 | -74.27 | -0.87 |
| 15 | 16.69 | 8.06 | 14.15 | -11.62 |
| 15-1 | 22.39 | 12.99 | 19.49 | -10.84 |
| 16 | -29.09 | -40.33 | -32.61 | -9.01 |
| thiophene-3-ol + OH | -24.47 | -43.28 | -28.23 | -1.45 |
| 17 | -55.68 | -64.25 | -53.89 | -11.69 |

**Table S2.** The thermodynamic parameters (in kcal mol^-1^) of all minimum stationary points at room temperature calculated at the B3LYP/6-311+ g(d,p) level.

| Saddle points | *ΔE^0^* | *ΔG^0^* | *ΔH^0^* | *TΔS^0^* | Imaginary frequency |
| --- | --- | --- | --- | --- | --- |
| Thiophene + HO_2_ | 0 | 0 | 0 | 0 |  |
| TS1 | 26.18 | 32.25 | 21.23 | -11.02 | 555.09i |
| TS2 | 20.98 | 27.06 | 16.04 | -11.02 | 512.55 i |
| TS3 | 43.25 | 48.47 | 38.29 | -10.17 | 801.58 i |
| TS4 | 67.84 | 74.12 | 62.89 | -11.23 | 1273.86 i |
| TS5 | 30.59 | 35.86 | 25.64 | -10.22 | 500.07 i |
| TS6 | 46.63 | 48.54 | 35.69 | -9.48 | 463.01 i |
| TS7 | 34.92 | 40.60 | 29.98 | 1.52 | 478.80 i |
| TS8 | -9.22 | 4.70 | 14.52 | -10.42 | 512.25i |
| TS8a | 19.83 | 24.64 | 14.88 | -9.75 | 310.06 i |
| TS9 | 35.92 | 41.69 | 30.97 | -9.12 | 312.54 i |
| TS10 | 23.99 | 30.53 | 19.04 | -11.48 | 873.30 i |
| TS11 | -28.06 | 39.67 | -33.01 | -10.37 | 212.20 i |
| TS12 | 18.85 | 23.13 | 13.90 | -9.23 | 408.89 i |
| TS13 | 15.52 | 19.42 | 10.58 | -8.85 | 833.30 i |
| TS14 | -29.21 | -23.89 | -34.16 | -9.39 | 127.34 i |
| TS15 | 32.78 | 40.33 | 27.83 | -12.49 | 408.89 i |
| TS16 | 40.33 | 46.38 | 35.38 | -10.99 | 1716.94 i |
| TS17 | -1.91 | 4.99 | -6.86 | -11.86 | 1910.17 i |

**Table S3.** The thermodynamic parameters (in kcal mol^-1^) of all saddle points at room temperature calculated at the B3LYP/6-311+ g(d,p) level.

|  |  |  | Eigenvalues of the Hessian matrix | | |  |
| --- | --- | --- | --- | --- | --- | --- |
| Species | Bond | ρ | λ1 | λ2 | λ3 | ∇^2^ρ |
| CR | H12-C4 | 0.01604 | -0.0175 | -0.0087 | 0.0677 | 0.0414 |
|  | H12-O11 | 0.3505 | -1.7564 | -1.7107 | 1.0192 | -2.4478‬ |
|  | O10-O11 | 0.3844 | -1.0004 | -0.9807 | 1.7664 | -0.2147 |
|  | C4-C3 | 0.3175 | -0.6628 | -0.5161 | 0.3056 | -0.8733 |
|  | RCP | 0.0394 | -0.0357 | 0.1296 | 0.1514 | 0.2453‬ |
| 1 | C3-O10 | 0.2367 | -0.4470 | -0.4169 | 0.4009 | -0.4630 |
|  | O10-O11 | 0.2635 | -0.6359 | 0.6007 | 1.3302 | 0.0935 |
|  | O11-H12 | 0.3674 | -1.8365 | -1.7606 | 1.0463 | -2.5508‬ |
|  | C3-H8 | 0.2796 | -0.7586 | -0.7429 | 0.5508 | -0.9504‬ |
|  | C3-C2 | 0.2570 | -0.5009 | -0.4855 | 0.3671 | -0.6192‬ |
| 2 | C1-O12 | 0.2482 | -0.489 | -0.4447 | 0.4093 | -0.5244‬ |
|  | O12-O10 | 0.2648 | -0.6380 | -0.6068 | 1.3344 | 0.0895 |
|  | O10-H11 | 0.3666 | -1.8360 | -1.7601 | 1.04587 | -2.5502 |
|  | C1-H6 | 0.2849 | -0.7853 | -0.7692 | 0.5702 | -0.984‬1 |
|  | C1-S5 | 0.1734 | -0.2693 | -0.2476 | 0.2654 | -0.2515 |
| 3 | S5-O10 | 0.2575 | -0.3799 | 0.3652 | 1.1009 | 0.3557 |
|  | O10-O11 | 0.0215 | -0.0236 | -0.0184 | 0.1567 | 0.1144‬ |
|  | O11-H12 | 0.3601 | -1.7620 | -0.914 | 1.0096 | -2.4499 |
|  | S5-C1 | 0.1964 | -0.3325 | -0.2943 | 0.2797 | -0.3470 |
|  | C1-H6 | 0.2830 | -0.7746 | -0.7568 | 0.5498 | -0.9816‬ |
| 5 | C1-O10 | 0.3643 | -0.4809 | -0.3180 | 0.4069 | -0.392‬0 |
|  | C2-O10 | 0.2277 | -0.4117 | -0.2300 | 0.3794 | -0.2623 |
|  | O10-H12 | 0.0246 | -0.0335 | -0.0332 | 0.1586 | 0.0918‬ |
|  | H12-O11 | 0.34796 | -1.726 | -1.6618 | 0.9877 | -2.401‬2 |
|  | RCP-epoxy | 0.2017 | -0.3691 | 0.2177 | 0.4862 | 0.3349 |
|  | C1-C2 | 0.2526 | -0.4869 | -0.4074 | 0.3587 | -0.5355 |
| 6 | C2-O10 | 0.2292 | -0.4276 | -0.2490 | 0.4105 | -0.2662 |
|  | C3-O10 | 0.2298 | -0.4250 | -0.2504 | 0.4043 | -0.2711 |
|  | C2-C3 | 0.2608 | -0.5113 | -0.4398 | 0.3581 | -0.5930 |
|  | RCP-epoxy | 0.1978 | -0.3643 | 0.2023 | 0.5162 | 0.3542 |
|  | RCP | 0.0096 | -0.0053 | 0.0077 | 0.0354 | 0.0378 |
|  | O10-H12 | 0.0100 | -0.0089 | -0.0062 | 0.0480 | 0.0328 |
|  | O11-H12 | 0.3549 | -1.7086 | -1.6779 | 0.9920 | -2.3945 |
|  | O11-S5 | 0.1518 | -0.2264 | -0.2264 | -0.2264 | -0.0323 |
| 7 | S5-C4 | 0.1502 | -0.2251 | -0.2100 | 0.2795 | -0.1556 |
|  | C4-O11 | 0.2848 | -0.5803 | -0.5519 | 0.5829 | -0.5493 |
|  | O11-H12 | 0.3533 | -1.755 | -1.7162 | 1.0133 | -2.4585 |
|  | C3-O10 | 0.0245 | -0.5694 | -0.5499 | 0.5505 | -0.5688 |
|  | O10-H12 | 0.0245 | -0.0290 | -0.0206 | 0.1434 | 0.0937 |
|  | C3-C4 | 0.2372 | -0.4602 | -0.4414 | 0.3851 | -0.5165 |
|  | RCP | 0.0231 | -0.0235 | 0.0275 | 0.1220 | 0.1260 |
| 8 | C4-S5 | 0.2793 | -0.7596 | -0.7500 | 0.5500 | -0.9596 |
|  | S-H6 | 0.0102 | -0.0079 | 0.0075 | 0.0451 | 0.0311 |
|  | C1-O12 | 0.3991 | -1.0188 | -0.9878 | 1.9486 | -0.0581 |
|  | O10-H11 | 0.34554 | -1.7187 | -1.6549 | 0.9870 | -2.3866 |
|  | H11-O12 | 0.0270 | -0.0379 | -0.0375 | 0.1701 | 0.0946 |
| 8a | C1-H6 | 0.2793 | -0.7596 | -0.7500 | 0.5500 | -1.1396 |
|  | C1-O12 | 0.3990 | -1.0188 | -0.9878 | 1.9486 | -0.0352 |
|  | O10-O11 | 0.0244 | -0.0231 | -0.0231 | 0.1562 | 0.1092 |
|  | O10-H11 | 0.3610 | -1.7589 | -1.6921 | 1.0074 | -2.4437 |
| 9 | C3-O10 | 0.3981 | -1.0143 | -0.9749 | 1.8474 | - 0.1416 |
|  | O10-O11 | 0.0254 | -0.0247 | -0.0216 | 0.1599 | 0.1137 |
|  | O11-C4 | 0.0089 | -0.0034 | -0.0017 | 0.0465 | 0.0414‬ |
|  | RCP | 0.2796 | -0.0029 | 0.0043 | 0.0414 | 0.0427‬ |
| 10 | C1-O10 | 0.2829 | -0.5723 | -0.4785 | 0.8116 | -0.2392‬ |
|  | C2-O10 | 0.2401 | -0.4798 | -0.4177 | 0.40009 | -0.4974 |
|  | C2-O11 | 0.2797 | -0.6045 | -0.5362 | 0.5381 | -0.6026 |
|  | C4-C3 | 0.2849 | -0.7853 | -0.7692 | 0.5702 | -0.984‬1 |
|  | RCP | 0.0172 | -0.0107 | 0.05202 | 0.0678 | 0.1089 |
| 11 | C3-O10 | 0.3772 | -0.9323 | -0.8819 | 1.5538 | -0.2604 |
|  | O10-H12 | 0.0265 | -0.0372 | -0.0358 | 0.1669 | 0.0938‬ |
|  | O11-H9 | 0.0103 | -0.0093 | -0.0085 | 0.0575 | 0.0397 |
|  | RCP | 0.0069 | -0.0052 | 0.0108 | 0.0294 | 0.0349 |
| 12 | C1-O10 | 0.2798 | -0.5766 | -0.5301 | 0.7397 | -0.367‬1 |
|  | H12-O10 | 0.0229 | -0.0310 | -0.0296 | 0.1480 | 0.0874 |
|  | C1-C2 | 0.3217 | -0.6804 | -0.5005 | 0.2873 | -0.8937 |
|  | H6-O10 | 0.3617 | -1.8081 | -1.7673 | 1.0409 | -2.5346‬ |
|  | RCP | 0.0385 | -0.0339 | 0.1261 | 0.1454 | 0.2376‬ |
| 13 | C1-O10 | 0.4116 | -1.0731 | -1.0230 | 2.03210 | -0.0640‬ |
|  | C1-C2 | 0.2468 | -0.4784 | 0.4546 | 0.3677 | -0.5652 |
|  | O10-H12 | 0.0257 | -0.0354 | -0.0350 | 0.1627 | 0.0923 |
|  | C2-H6 | 0.2710 | -0.7094 | -0.6989 | 0.5137 | -0.8944 |
|  | RCP | 0.0328 | -0.0302 | 0.1053 | 0.1261 | 0.2013 |
| 14 | C1-O10 | 0.4016 | -1.0396 | -0.9722 | 1.8987 | -0.1131 |
|  | O10-H12 | 0.0222 | -0.0292 | -0.0281 | 0.1389 | 0.0815 |
|  | C2-H7 | 0.2835 | -0.7816 | -0.7670 | 0.5573 | -0.9913 |
|  | H7-O11 | 0.0084 | -0.0071 | -0.0067 | 0.0447 | 0.0309 |
|  | RCP | 0.0058 | -0.0043 | 0.0092 | 0.0243 | 0.0292 |
| 15 | O11-S5 | 0.1737 | -0.2603 | -0.2496 | 0.3451 | -0.1647 |
|  | O11-H12 | 0.3505 | -1.7188 | -1.6836 | 0.9932 | -2.4092 |
|  | H12-O10 | 0.0083 | -0.0064 | -0.0014 | 0.0350 | 0.0271 |
|  | C2-O10 | 0.2907 | -0.5826 | -0.5698 | 0.5009 | -0.6515 |
|  | C1-C2 | 0.3438 | -0.7306 | -0.5509 | 0.2743 | -1.0072 |
|  | RCP | 0.0366 | -0.0302 | 0.11873 | 0.1401 | 0.2285‬ |
| 15-1 | S5-O11 | 0.1409 | -0.2075 | -0.2005 | 0.4295 | 0.0213 |
|  | C1-S5 | 0.2422 | -0.4038 | -0.2927 | 0.1848 | -0.5117 |
|  | C2-O10 | 0.2939 | -0.60592 | -0.5693 | 0.6289 | -0.5463 |
|  | RCP | 0.03625 | -0.0303 | 0.1134 | 0.1440 | 0.2271 |
| 16 | S5-O11 | 0.0397 | -0.0415 | -0.0368 | 0.1969 | 0.1184 |
|  | O10-C2 | 0.2864 | -0.5868 | -0.5712 | 0.7829 | -0.3752 |
|  | H7-O10 | 0.3662 | -1.8146 | -1.7747 | -2.5488 | -2.3625 |
|  | C2-C3 | 0.3222 | -0.6942 | -0.5120 | 0.3087 | -0.7472 |
| 17 | C4-O11 | 0.2567 | -0.4906 | -0.4701 | 0.5307 | -1.1382 |
|  | C3-O10 | 0.2848 | -0.5813 | -0.5273 | 0.8024 | -0.3063 |
|  | H12-O11 | 0.3620 | -1.7682 | -1.7257 | 1.0145 | -2.4795 |
|  | C1-C2 | 0.3063 | -0.6342 | -0.5102 | 0.3174 | -0.8269 |
| TS1 | C3-O10 | 0.1062 | 0.1651 | -0.1474 | 0.4405 | 0.1280 |
|  | O10-O11 | 0.3000 | -0.7317 | -0.7188 | 1.4752 | 0.0247 |
|  | O11-H12 | 0.3678 | -1.8174 | -1.7484 | 1.0368 | -2.5289 |
|  | C3-H8 | 0.2862 | -0.7813 | -0.7797 | 0.5596 | -1.0014 |
|  | C3-C2 | 0.2939 | -0.5943 | -0.5188 | 0.3390 | -0.7741 |
| TS2 | C1-O12 | 0.0882 | -0.1302 | -0.1123 | 0.3933 | 0.1507 |
|  | O12-O10 | 0.3104 | -0.7652 | -0.7508 | 1.5130 | -0.0030 |
|  | O10-H11 | 0.3676 | -1.8186 | -1.7515 | 1.0377 | -2.5325 |
|  | C1-H6 | 0.2882 | -0.7950 | -0.7907 | 0.5721 | -1.0136 |
|  | C1-S5 | 0.1996 | -0.3226 | -0.2764 | 0.2423 | -0.3567 |
| TS3 | S5-O10 | 0.1726 | -0.2584 | -0.2525 | 0.4691 | -0.0419 |
|  | O10-O11 | 0.1338 | -0.2469 | -0.2397 | 0.8902 | 0.4035 |
|  | O11-H12 | 0.3659 | -1.7744 | -1.7148 | 1.0221 | -2.4671 |
|  | S5-C1 | 0.2049 | -0.3424 | -0.2965 | 0.2654 | -0.3734 |
|  | C1-H6 | 0.2834 | -0.7777 | -0.7538 | 0.5500 | -0.9814 |
| TS4 | C3-O10 | 0.2059 | -0.3642 | -0.3441 | 0.3997 | -0.3086 |
|  | O10-O11 | 0.1691 | -0.3256 | -0.2149 | 0.9739 | 0.4335 |
|  | O10-H11 | 0.2720 | -1.2064 | -1.1342 | 0.8201 | -1.5205 |
|  | C3-H8 | 0.2833 | -0.7795 | -0.7620 | 0.5639 | -0.9776 |
|  | C3-C2 | 0.2662 | -0.5171 | -0.4849 | 0.3581 | -0.6439 |
| TS5 | C2-O10 | 0.2723 | -0.5219 | -0.4968 | 0.4596 | -0.5590 |
|  | C2-H7 | 0.2816 | -0.7669 | -0.7555 | 0.5538 | -0.9686 |
|  | O10-O11 | 0.0895 | -0.1495 | -0.1336 | 0.6134 | 0.3303 |
|  | H12-O11 | 0.3641 | -1.7637 | -1.7026 | 1.0174 | -2.4490 |
|  | C1-C2 | 0.2622 | -0.5076 | -0.4794 | 0.3550 | -0.6320 |
| TS6 | C2-O12 | 0.2657 | -0.5116 | -0.4395 | 0.4184 | -0.5327 |
|  | C2-C3 | 0.2484 | -0.4812 | -0.4287 | 0.3545 | -0.5554 |
|  | O10-O12 | 0.0461 | -0.0606 | -0.0536 | 0.3242 | 0.2100 |
|  | O11-H12 | 0.3628 | -1.7439 | -1.6837 | 1.0084 | -2.4192 |
|  | C2-H8 | 0.2851 | -0.7849 | -0.7694 | 0.5610 | -0.9933 |
| TS7 | C3-O10 | 0.3149 | -0.6752 | -0.6539 | 0.6419 | -0.6872 |
|  | O10-O11 | 0.0813 | -0.1283 | -0.1178 | 0.5542 | 0.3081 |
|  | O11-H12 | 0.3650 | -1.7662 | -1.7119 | 1.0231 | -2.4550 |
|  | C3-C4 | 0.2615 | -0.5197 | -0.4756 | 0.3685 | -0.6268 |
|  | C3-O10 | 0.3149 | -0.6752 | -0.6539 | 0.6419 | -0.6872 |
|  | O10-O11 | 0.0813 | -0.1283 | -0.1178 | 0.5542 | 0.3081 |
| TS8 | C1-H6 | 0.2857 | -0.7898 | -0.7802 | 0.5733 | -0.9968 |
|  | S5-C1 | 0.1387 | -0.2006 | -0.1941 | 0.2794 | -0.1154 |
|  | C1-O12 | 0.3155 | -0.6668 | -0.6216 | 0.6803 | -0.6081 |
|  | O12-O10 | 0.0427 | -0.0534 | -0.0488 | 0.2970 | 0.1949 |
|  | O10-H11 | 0.3624 | -1.7518 | -1.6903 | 1.0100 | -2.4322 |
|  | C1-S5 | 0.1248 | -0.1745 | -0.1689 | 0.2697 | -0.0737 |
| TS8a | C1-H6 | 0.2827 | -0.7782 | -0.7688 | 0.5725 | -0.9744 |
|  | C1-O12 | 0.3224 | -0.7054 | -0.6724 | 0.8278 | -0.5500 |
|  | O10-H11 | 0.3642 | -1.7654 | -1.7061 | 1.0194 | -2.4520 |
|  | O10-O12 | 0.0875 | -0.1445 | -0.1310 | 0.6057 | 0.3302 |
| TS9 | C3-O10 | 0.3165 | -0.6829 | -0.6509 | 0.5141 | -0.8197 |
|  | O10-O11 | 0.0366 | -0.0383 | -0.0378 | 0.2277 | 0.1516 |
|  | O11-H9 | 0.0187 | -0.0200 | -0.0145 | 0.1126 | 0.0781 |
|  | RCP | 0.0154 | -0.0107 | 0.0166 | 0.0696 | 0.0755 |
| TS10 | C1-O10 | 0.2822 | -0.5570 | -0.4938 | 0.5700 | -0.4807 |
|  | C2-O10 | 0.2538 | -0.4644 | -0.4504 | 0.4199 | -0.4949 |
|  | C2-O11 | 0.0722 | -0.0996 | -0.0803 | 0.3384 | 0.1585 |
|  | C4-C3 | 0.3373 | -0.7234 | -0.5318 | 0.2811 | -0.9742 |
|  | RCP | 0.0311 | -0.0281 | 0.0878 | 0.1181 | 0.1778 |
| TS11 | C3-O10 | 0.4058 | -1.0459 | -0.9951 | 1.9142 | -0.1267 |
|  | O10-H12 | 0.3570 | -1.7246 | -1.6655 | 0.9873 | -2.4028 |
|  | O11-H8 | 0.0707 | -0.1375 | -0.1293 | 0.4205 | 0.1537 |
|  | C4-C3 | 0.2443 | -0.4771 | -0.4516 | 0.3809 | -0.5478 |
| TS12 | C1-O10 | 0.3007 | -0.6265 | -0.5511 | 0.4697 | -0.7079 |
|  | H12-O10 | 0.0339 | -0.0508 | -0.0501 | 0.2171 | 0.1163 |
|  | O11-H12 | 0.3417 | -1.7014 | -1.6394 | 0.9862 | -2.3547 |
|  | C1-H6 | 0.1996 | -0.4924 | -0.3929 | 0.4425 | -0.4428 |
|  | C1-C2 | 0.2974 | -0.6178 | -0.5114 | 0.3383 | -0.7909 |
| TS13 | C1-O12 | 0.3895 | -0.9328 | -0.9108 | 1.4075 | -0.4361 |
|  | C1-C2 | 0.2731 | -0.5525 | -0.4822 | 0.3699 | -0.6648 |
|  | O10-H11 | 0.3405 | -1.6963 | -1.6350 | 0.9809 | -2.3503 |
|  | C1-H6 | 0.1864 | -0.4163 | -0.3351 | 0.3817 | -0.3697 |
|  | O12-H11 | 0.0329 | -0.0493 | -0.0481 | 0.2146 | 0.1171 |
| TS14 | C1-O10 | 0.4195 | -1.1052 | -1.0478 | -1.0478 | -0.0157 |
|  | O11-H12 | 0.3568 | -1.7271 | -1.6653 | 0.9869 | -2.4055 |
|  | C2-H6 | 0.2444 | -0.6268 | -0.6193 | 0.5110 | -0.7351 |
|  | C2-H7 | 0.2725 | -0.7174 | -0.7044 | 0.5176 | -0.9042 |
|  | H6-O11 | 0.0500 | -0.0847 | -0.0788 | 0.3104 | 0.1469 |
| TS15 | O11-S5 | 0.1779 | -0.2615 | -0.2589 | 0.3307 | -0.1898 |
|  | O11-H12 | 0.3447 | -1.7039 | -1.6728 | 0.9912 | -2.3855 |
|  | H12-O10 | 0.0190 | -0.0212 | -0.0206 | 0.1016 | 0.0598 |
|  | C2-O10 | 0.2604 | -0.5051 | -0.4265 | 0.4121 | -0.5194 |
|  | RCP | 0.0133 | -0.0060 | 0.0199 | 0.0436 | 0.0574 |
| TS16 | S5-O11 | 0.1130 | -0.1591 | -0.1524 | 0.4420 | 0.1305 |
|  | O10-C2 | 0.2917 | -0.5992 | -0.5224 | 0.4772 | -0.6443 |
|  | H12-O11 | 0.3587 | -1.6889 | -1.6522 | 0.9912 | -2.3498 |
|  | C2-H7 | 0.1894 | -0.4443 | -0.3185 | 0.4010 | -0.3618 |
| TS17 | C4-O11 | 0.2665 | -0.5369 | -0.4956 | 0.4344 | -0.5980 |
|  | O10-H8 | 0.1756 | -0.4158 | -0.0617 | 0.5344 | 0.0569 |
|  | C3-O10 | 0.2788 | -0.5504 | -0.4719 | 0.4467 | -0.5756 |
|  | C3-H8 | 0.1776 | -0.4023 | -0.1386 | 0.3719 | -0.1691 |
|  | O11-H12 | 0.3588 | -1.7622 | -1.7169 | 1.0095 | -2.4697 |

**Table S4.** Topological parameters of the line critical points and ring critical points (in atomic unit) of all stationary points calculated at the B3LYP/6-311+ g(d,p) level for the C_4_H_4_S + HO_2_ reaction.

| *T/K* | *k_b1_* | *k_b2_* | *k_b3_* | *k_b4_* | *k_b5_* | *k_b6_* | *k_b7_* | *k_b8_* | *k_b8a_* |
| --- | --- | --- | --- | --- | --- | --- | --- | --- | --- |
| 300 | 9.43E-05 | 8.43E-02 | 2.04E-17 | 9.33E-33 | 1.22E-06 | 3.64E-20 | 9.33E-33 | 1.22E-06 | 1.31E-07 |
| 400 | 5.77E-02 | 9.53E+00 | 2.49E-11 | 2.53E-23 | 1.96E-03 | 1.72E-13 | 2.53E-23 | 1.96E-03 | 1.05E-04 |
| 500 | 3.22E+00 | 1.92E+02 | 1.43E-07 | 2.08E-17 | 2.30E-01 | 2.65E-09 | 2.08E-17 | 2.29E-01 | 1.18E-02 |
| 600 | 5.27E+01 | 1.59E+03 | 5.29E-05 | 2.23E-13 | 6.56E+00 | 2.09E-06 | 2.23E-13 | 6.53E+00 | 4.66E-01 |
| 700 | 4.20E+02 | 7.77E+03 | 3.97E-03 | 1.89E-10 | 7.86E+01 | 2.85E-04 | 1.89E-10 | 7.81E+01 | 9.13E+00 |
| 800 | 2.11E+03 | 2.71E+04 | 1.08E-01 | 3.19E-08 | 5.33E+02 | 1.25E-02 | 3.19E-08 | 5.29E+02 | 1.09E+02 |
| 900 | 7.75E+03 | 7.47E+04 | 1.49E+00 | 1.82E-06 | 2.44E+03 | 2.52E-01 | 1.82E-06 | 2.42E+03 | 9.22E+02 |
| 1000 | 2.27E+04 | 1.74E+05 | 1.26E+01 | 4.85E-05 | 8.43E+03 | 2.92E+00 | 4.85E-05 | 8.34E+03 | 6.38E+03 |
| 1100 | 5.63E+04 | 3.59E+05 | 7.48E+01 | 7.33E-04 | 2.37E+04 | 2.22E+01 | 7.33E-04 | 2.34E+04 | 3.75E+04 |
| 1200 | 1.23E+05 | 6.70E+05 | 3.38E+02 | 7.25E-03 | 5.71E+04 | 1.21E+02 | 7.25E-03 | 5.63E+04 | 1.72E+05 |
| 1300 | 2.43E+05 | 1.16E+06 | 1.23E+03 | 5.16E-02 | 1.22E+05 | 4.98E+02 | 5.16E-02 | 1.20E+05 | 4.72E+05 |
| 1400 | 4.42E+05 | 1.89E+06 | 3.82E+03 | 2.82E-01 | 2.36E+05 | 1.62E+03 | 2.82E-01 | 2.31E+05 | 8.98E+05 |
| 1500 | 7.55E+05 | 2.92E+06 | 1.03E+04 | 1.25E+00 | 4.21E+05 | 4.35E+03 | 1.25E+00 | 4.13E+05 | 1.44E+06 |
| 1600 | 1.22E+06 | 4.34E+06 | 2.49E+04 | 4.69E+00 | 7.05E+05 | 9.95E+03 | 4.68E+00 | 6.91E+05 | 2.16E+06 |
| 1700 | 1.89E+06 | 6.22E+06 | 5.50E+04 | 1.52E+01 | 1.12E+06 | 2.00E+04 | 1.52E+01 | 1.09E+06 | 3.11E+06 |
| 1800 | 2.81E+06 | 8.65E+06 | 1.12E+05 | 4.37E+01 | 1.70E+06 | 3.65E+04 | 4.36E+01 | 1.66E+06 | 4.32E+06 |
| 1900 | 4.04E+06 | 1.17E+07 | 2.14E+05 | 1.13E+02 | 2.48E+06 | 6.14E+04 | 1.13E+02 | 2.42E+06 | 5.86E+06 |
| 2000 | 5.65E+06 | 1.55E+07 | 3.86E+05 | 2.70E+02 | 3.50E+06 | 9.71E+04 | 2.70E+02 | 3.43E+06 | 7.76E+06 |
| 2100 | 7.71E+06 | 2.02E+07 | 6.64E+05 | 5.97E+02 | 4.83E+06 | 1.46E+05 | 5.97E+02 | 4.72E+06 | 1.01E+07 |
| 2200 | 1.03E+07 | 2.58E+07 | 1.09E+06 | 1.24E+03 | 6.49E+06 | 2.11E+05 | 1.24E+03 | 6.34E+06 | 1.29E+07 |
| 2300 | 1.35E+07 | 3.24E+07 | 1.73E+06 | 2.42E+03 | 8.55E+06 | 2.95E+05 | 2.42E+03 | 8.36E+06 | 1.62E+07 |
| 2400 | 1.74E+07 | 4.02E+07 | 2.66E+06 | 4.50E+03 | 1.11E+07 | 4.00E+05 | 4.50E+03 | 1.08E+07 | 2.01E+07 |
| 2500 | 2.20E+07 | 4.93E+07 | 3.97E+06 | 8.01E+03 | 1.41E+07 | 5.31E+05 | 8.01E+03 | 1.38E+07 | 2.46E+07 |
| 2600 | 2.76E+07 | 5.97E+07 | 5.76E+06 | 1.37E+04 | 1.77E+07 | 6.89E+05 | 1.37E+04 | 1.73E+07 | 2.99E+07 |
| 2700 | 3.41E+07 | 7.17E+07 | 8.18E+06 | 2.27E+04 | 2.19E+07 | 8.79E+05 | 2.26E+04 | 2.14E+07 | 3.58E+07 |
| 2800 | 4.16E+07 | 8.53E+07 | 1.14E+07 | 3.63E+04 | 2.68E+07 | 1.11E+06 | 3.62E+04 | 2.62E+07 | 4.26E+07 |
| 2900 | 5.04E+07 | 1.01E+08 | 1.55E+07 | 5.64E+04 | 3.25E+07 | 1.37E+06 | 5.64E+04 | 3.18E+07 | 5.02E+07 |
| 3000 | 6.04E+07 | 1.18E+08 | 2.08E+07 | 8.55E+04 | 3.91E+07 | 1.68E+06 | 8.54E+04 | 3.81E+07 | 5.87E+07 |
| *T/K* | *k_b9_* | *k_b10_* | *k_b11_* | *k_b12_* | *k_b13_* | *k_b14_* | *k_b15_* | *k_b16_* | *k_b17_* |
| 300 | 5.77E-13 | 1.20E-06 | 5.77E-13 | 3.49E-10 | 1.22E-06 | 1.22E-06 | 3.64E-20 | 2.06E-20 | 9.33E-33 |
| 400 | 1.97E-08 | 1.87E-03 | 1.97E-08 | 4.54E-06 | 1.96E-03 | 1.96E-03 | 1.72E-13 | 7.16E-14 | 2.53E-23 |
| 500 | 1.33E-05 | 2.11E-01 | 1.33E-05 | 1.98E-03 | 2.29E-01 | 2.29E-01 | 2.65E-09 | 9.16E-10 | 2.08E-17 |
| 600 | 1.30E-03 | 5.79E+00 | 1.30E-03 | 1.49E-01 | 6.52E+00 | 6.52E+00 | 2.09E-06 | 6.36E-07 | 2.23E-13 |
| 700 | 4.14E-02 | 6.69E+01 | 4.14E-02 | 3.72E+00 | 7.80E+01 | 7.80E+01 | 2.85E-04 | 7.75E-05 | 1.89E-10 |
| 800 | 6.42E-01 | 4.38E+02 | 6.42E-01 | 4.39E+01 | 5.28E+02 | 5.28E+02 | 1.25E-02 | 3.08E-03 | 3.19E-08 |
| 900 | 5.96E+00 | 1.94E+03 | 5.96E+00 | 3.00E+02 | 2.41E+03 | 2.41E+03 | 2.52E-01 | 5.65E-02 | 1.82E-06 |
| 1000 | 3.78E+01 | 6.47E+03 | 3.78E+01 | 1.34E+03 | 8.32E+03 | 8.32E+03 | 2.92E+00 | 5.98E-01 | 4.85E-05 |
| 1100 | 1.78E+02 | 1.76E+04 | 1.78E+02 | 4.45E+03 | 2.34E+04 | 2.34E+04 | 2.22E+01 | 4.18E+00 | 7.33E-04 |
| 1200 | 6.53E+02 | 4.12E+04 | 6.53E+02 | 1.26E+04 | 5.61E+04 | 5.61E+04 | 1.21E+02 | 2.12E+01 | 7.25E-03 |
| 1300 | 1.98E+03 | 8.56E+04 | 1.98E+03 | 3.35E+04 | 1.19E+05 | 1.19E+05 | 4.98E+02 | 8.26E+01 | 5.16E-02 |
| 1400 | 5.09E+03 | 1.62E+05 | 5.09E+03 | 7.87E+04 | 2.30E+05 | 2.30E+05 | 1.62E+03 | 2.58E+02 | 2.82E-01 |
| 1500 | 1.14E+04 | 2.84E+05 | 1.14E+04 | 1.63E+05 | 4.11E+05 | 4.11E+05 | 4.35E+03 | 6.69E+02 | 1.25E+00 |
| 1600 | 2.25E+04 | 4.70E+05 | 2.25E+04 | 3.03E+05 | 6.88E+05 | 6.88E+05 | 9.95E+03 | 1.49E+03 | 4.68E+00 |
| 1700 | 4.08E+04 | 7.39E+05 | 4.08E+04 | 5.20E+05 | 1.09E+06 | 1.09E+06 | 2.00E+04 | 2.95E+03 | 1.52E+01 |
| 1800 | 6.86E+04 | 1.11E+06 | 6.86E+04 | 8.33E+05 | 1.65E+06 | 1.65E+06 | 3.65E+04 | 5.31E+03 | 4.36E+01 |
| 1900 | 1.09E+05 | 1.62E+06 | 1.09E+05 | 1.27E+06 | 2.41E+06 | 2.41E+06 | 6.14E+04 | 8.85E+03 | 1.13E+02 |
| 2000 | 1.64E+05 | 2.28E+06 | 1.64E+05 | 1.85E+06 | 3.41E+06 | 3.41E+06 | 9.71E+04 | 1.39E+04 | 2.70E+02 |
| 2100 | 2.37E+05 | 3.12E+06 | 2.37E+05 | 2.62E+06 | 4.70E+06 | 4.70E+06 | 1.46E+05 | 2.07E+04 | 5.97E+02 |
| 2200 | 3.32E+05 | 4.19E+06 | 3.32E+05 | 3.59E+06 | 6.31E+06 | 6.31E+06 | 2.11E+05 | 2.98E+04 | 1.24E+03 |
| 2300 | 4.52E+05 | 5.51E+06 | 4.52E+05 | 4.80E+06 | 8.31E+06 | 8.31E+06 | 2.95E+05 | 4.14E+04 | 2.42E+03 |
| 2400 | 6.01E+05 | 7.11E+06 | 6.01E+05 | 6.30E+06 | 1.08E+07 | 1.08E+07 | 4.00E+05 | 5.60E+04 | 4.50E+03 |
| 2500 | 7.83E+05 | 9.04E+06 | 7.83E+05 | 8.11E+06 | 1.37E+07 | 1.37E+07 | 5.31E+05 | 7.40E+04 | 8.01E+03 |
| 2600 | 1.00E+06 | 1.13E+07 | 1.00E+06 | 1.03E+07 | 1.72E+07 | 1.72E+07 | 6.89E+05 | 9.59E+04 | 1.37E+04 |
| 2700 | 1.26E+06 | 1.40E+07 | 1.26E+06 | 1.28E+07 | 2.13E+07 | 2.13E+07 | 8.79E+05 | 1.22E+05 | 2.26E+04 |
| 2800 | 1.57E+06 | 1.72E+07 | 1.57E+06 | 1.58E+07 | 2.61E+07 | 2.61E+07 | 1.11E+06 | 1.53E+05 | 3.62E+04 |
| 2900 | 1.93E+06 | 2.08E+07 | 1.93E+06 | 1.93E+07 | 3.16E+07 | 3.16E+07 | 1.37E+06 | 1.89E+05 | 5.64E+04 |
| 3000 | 2.34E+06 | 2.49E+07 | 2.34E+06 | 2.33E+07 | 3.79E+07 | 3.79E+07 | 1.68E+06 | 2.32E+05 | 8.54E+04 |

**Table S5.** The calculated bimolecular rate constants (L mol^-1^ s^−1^) for production of all minimum stationary points the computed PES at the BD(T)/6-31+g(d,p) level.

| *T/K* | *k_1_* | *k_-1_* | *k_2_* | *k_-2_* | *k_3_* | *k_-3_* | *k_4_* | *k_-4_* | *k_5_* |
| --- | --- | --- | --- | --- | --- | --- | --- | --- | --- |
| 300 | 9.48E-06 | 7.86E+08 | 7.22E-03 | 1.19E+07 | 2.52E-18 | 4.05E-06 | 1.07E-33 | 9.04E-03 | 1.03E+07 |
| 400 | 5.47E-02 | 6.81E+08 | 7.96E+00 | 3.34E+07 | 2.78E-11 | 5.51E-02 | 2.61E-23 | 1.93E-01 | 2.40E+07 |
| 500 | 1.02E+01 | 3.64E+08 | 5.47E+02 | 4.64E+07 | 5.20E-07 | 1.75E+01 | 7.02E-17 | 1.33E+00 | 2.79E+07 |
| 600 | 3.39E+02 | 1.66E+08 | 9.27E+03 | 4.00E+07 | 3.85E-04 | 8.27E+02 | 1.51E-12 | 5.62E+00 | 2.36E+07 |
| 700 | 4.16E+03 | 6.92E+07 | 6.96E+04 | 2.21E+07 | 4.43E-02 | 1.28E+04 | 1.96E-09 | 1.71E+01 | 1.59E+07 |
| 800 | 2.72E+04 | 3.14E+07 | 3.09E+05 | 1.24E+07 | 1.58E+00 | 9.61E+04 | 4.34E-07 | 4.06E+01 | 1.06E+07 |
| 900 | 1.16E+05 | 1.43E+07 | 9.55E+05 | 6.63E+06 | 2.57E+01 | 4.33E+05 | 2.94E-05 | 7.00E+01 | 6.55E+06 |
| 1000 | 3.62E+05 | 6.50E+06 | 2.27E+06 | 3.32E+06 | 2.40E+02 | 1.34E+06 | 8.65E-04 | 1.09E+02 | 3.84E+06 |
| 1100 | 8.97E+05 | 3.01E+06 | 4.49E+06 | 1.75E+06 | 1.49E+03 | 3.18E+06 | 1.39E-02 | 1.46E+02 | 2.19E+06 |
| 1200 | 1.86E+06 | 1.28E+06 | 7.68E+06 | 1.01E+06 | 6.82E+03 | 6.19E+06 | 1.40E-01 | 1.63E+02 | 1.11E+06 |
| 1300 | 3.37E+06 | 5.64E+05 | 1.20E+07 | 9.23E+05 | 2.44E+04 | 1.06E+07 | 1.00E+00 | 1.63E+02 | 5.68E+05 |
| 1400 | 5.48E+06 | 2.27E+05 | 1.70E+07 | 1.25E+06 | 7.14E+04 | 1.59E+07 | 5.40E+00 | 1.33E+02 | 2.58E+05 |
| 1500 | 8.17E+06 | 9.02E+04 | 2.22E+07 | 1.97E+06 | 1.77E+05 | 2.13E+07 | 2.33E+01 | 9.52E+01 | 1.14E+05 |
| 1600 | 1.16E+07 | 3.93E+04 | 2.90E+07 | 3.14E+06 | 3.84E+05 | 2.86E+07 | 8.40E+01 | 6.52E+01 | 5.36E+04 |
| 1700 | 1.51E+07 | 1.64E+04 | 3.35E+07 | 4.79E+06 | 7.38E+05 | 3.33E+07 | 2.61E+02 | 3.86E+01 | 2.39E+04 |
| 1800 | 1.96E+07 | 8.05E+03 | 4.04E+07 | 7.01E+06 | 1.29E+06 | 4.07E+07 | 7.13E+02 | 2.31E+01 | 1.23E+04 |
| 1900 | 2.34E+07 | 5.76E+03 | 4.52E+07 | 9.87E+06 | 2.08E+06 | 4.51E+07 | 1.75E+03 | 1.27E+01 | 9.13E+03 |
| 2000 | 2.68E+07 | 1.09E+04 | 5.21E+07 | 1.34E+07 | 3.13E+06 | 5.39E+07 | 3.94E+03 | 8.71E+00 | 1.78E+04 |
| 2100 | 3.15E+07 | 3.10E+04 | 5.80E+07 | 1.77E+07 | 4.49E+06 | 6.02E+07 | 8.18E+03 | 1.35E+01 | 5.18E+04 |
| 2200 | 3.48E+07 | 8.38E+04 | 6.75E+07 | 2.28E+07 | 6.09E+06 | 7.50E+07 | 1.59E+04 | 3.61E+01 | 1.43E+05 |
| 2300 | 3.90E+07 | 2.02E+05 | 7.32E+07 | 2.88E+07 | 8.06E+06 | 8.21E+07 | 2.90E+04 | 9.93E+01 | 3.49E+05 |
| 2400 | 4.32E+07 | 4.33E+05 | 8.60E+07 | 3.55E+07 | 1.01E+07 | 1.02E+08 | 5.01E+04 | 2.45E+02 | 7.59E+05 |
| 2500 | 4.92E+07 | 8.43E+05 | 9.99E+07 | 4.31E+07 | 1.21E+07 | 1.19E+08 | 8.24E+04 | 5.41E+02 | 1.49E+06 |
| 2600 | 5.28E+07 | 1.50E+06 | 6.23E+07 | 5.14E+07 | 1.46E+07 | 1.29E+08 | 1.30E+05 | 1.08E+03 | 2.69E+06 |
| 2700 | 5.30E+07 | 2.50E+06 | 5.30E+07 | 6.04E+07 | 1.63E+07 | 1.42E+08 | 1.96E+05 | 1.99E+03 | 4.50E+06 |
| 2800 | 6.67E+07 | 3.89E+06 | 8.34E+07 | 6.99E+07 | 1.93E+07 | 1.16E+08 | 2.85E+05 | 3.39E+03 | 7.05E+06 |
| 2900 | 7.14E+07 | 5.73E+06 | 7.14E+07 | 7.98E+07 | 2.06E+07 | 9.50E+07 | 3.99E+05 | 5.44E+03 | 1.04E+07 |
| 3000 | 6.16E+07 | 7.96E+06 | 6.16E+07 | 9.00E+07 | 2.18E+07 | 7.82E+07 | 5.42E+05 | 8.23E+03 | 1.46E+07 |
| *T/K* | *k_-5_* | *k_6_* | *k_-6_* | *k_7_* | *k_-7_* | *k_8_* | *k_-8_* | *k_8a_* | *k_-8a_* |
| 300 | 8.43E+01 | 3.03E-07 | 6.73E-10 | 1.04E+07 | 3.44E+01 | 1.65E+05 | 1.42E+01 | 2.66E+06 | 4.14E+00 |
| 400 | 2.24E+03 | 2.03E-03 | 5.31E-05 | 1.05E+07 | 1.52E+02 | 1.46E+06 | 6.70E+02 | 1.13E+07 | 1.24E+02 |
| 500 | 1.50E+04 | 2.99E-01 | 3.65E-02 | 1.01E+07 | 4.85E+02 | 4.89E+06 | 7.95E+03 | 2.09E+07 | 1.29E+03 |
| 600 | 4.26E+04 | 6.58E+00 | 1.97E+00 | 9.42E+06 | 1.47E+03 | 8.54E+06 | 3.78E+04 | 2.29E+07 | 6.73E+03 |
| 700 | 6.68E+04 | 4.69E+01 | 2.30E+01 | 8.49E+06 | 4.19E+03 | 9.15E+06 | 9.45E+04 | 1.57E+07 | 1.84E+04 |
| 800 | 8.13E+04 | 1.85E+02 | 1.18E+02 | 7.39E+06 | 1.10E+04 | 8.03E+06 | 1.72E+05 | 1.06E+07 | 4.29E+04 |
| 900 | 7.96E+04 | 4.64E+02 | 3.38E+02 | 5.42E+06 | 2.28E+04 | 5.89E+06 | 2.35E+05 | 6.63E+06 | 8.29E+04 |
| 1000 | 6.71E+04 | 8.35E+02 | 6.53E+02 | 3.99E+06 | 4.42E+04 | 3.80E+06 | 2.68E+05 | 3.80E+06 | 1.44E+05 |
| 1100 | 5.20E+04 | 1.19E+03 | 9.59E+02 | 2.76E+06 | 7s.38E+04 | 2.32E+06 | 2.71E+05 | 2.24E+06 | 2.62E+05 |
| 1200 | 3.43E+04 | 1.26E+03 | 1.04E+03 | 1.70E+06 | 9.82E+04 | 1.25E+06 | 2.05E+05 | 1.43E+06 | 4.95E+05 |
| 1300 | 2.14E+04 | 1.16E+03 | 9.68E+02 | 1.01E+06 | 1.10E+05 | 6.50E+05 | 1.21E+05 | 1.42E+06 | 9.75E+05 |
| 1400 | 1.13E+04 | 8.35E+02 | 7.03E+02 | 5.43E+05 | 9.72E+04 | 2.97E+05 | 5.68E+04 | 2.05E+06 | 1.86E+06 |
| 1500 | 5.64E+03 | 5.23E+02 | 4.43E+02 | 2.81E+05 | 7.20E+04 | 1.31E+05 | 2.50E+04 | 3.43E+06 | 3.36E+06 |
| 1600 | 2.93E+03 | 3.23E+02 | 2.75E+02 | 1.52E+05 | 5.01E+04 | 6.20E+04 | 1.17E+04 | 5.73E+06 | 5.70E+06 |
| 1700 | 1.40E+03 | 1.76E+02 | 1.50E+02 | 7.61E+04 | 2.99E+04 | 2.75E+04 | 5.15E+03 | 9.15E+06 | 9.13E+06 |
| 1800 | 7.58E+02 | 1.06E+02 | 9.07E+01 | 4.09E+04 | 1.85E+04 | 1.41E+04 | 2.60E+03 | 1.39E+07 | 1.39E+07 |
| 1900 | 5.86E+02 | 8.90E+01 | 7.63E+01 | 2.26E+04 | 1.27E+04 | 1.04E+04 | 1.91E+03 | 2.03E+07 | 2.03E+07 |
| 2000 | 1.18E+03 | 1.90E+02 | 1.64E+02 | 2.02E+04 | 1.62E+04 | 2.02E+04 | 3.66E+03 | 2.85E+07 | 2.85E+07 |
| 2100 | 3.50E+03 | 5.98E+02 | 5.15E+02 | 3.82E+04 | 3.65E+04 | 5.84E+04 | 1.04E+04 | 3.88E+07 | 3.88E+07 |
| 2200 | 9.81E+03 | 1.75E+03 | 1.51E+03 | 9.34E+04 | 9.10E+04 | 1.60E+05 | 2.81E+04 | 5.13E+07 | 5.13E+07 |
| 2300 | 2.43E+04 | 4.50E+03 | 3.88E+03 | 2.23E+05 | 2.12E+05 | 3.88E+05 | 6.71E+04 | 6.62E+07 | 6.62E+07 |
| 2400 | 5.33E+04 | 1.02E+04 | 8.81E+03 | 4.86E+05 | 4.50E+05 | 8.41E+05 | 1.43E+05 | 8.36E+07 | 8.35E+07 |
| 2500 | 1.06E+05 | 2.08E+04 | 1.79E+04 | 9.71E+05 | 8.73E+05 | 1.64E+06 | 2.75E+05 | 1.03E+08 | 1.03E+08 |
| 2600 | 1.92E+05 | 3.86E+04 | 3.33E+04 | 1.79E+06 | 1.56E+06 | 2.95E+06 | 4.86E+05 | 1.26E+08 | 1.26E+08 |
| 2700 | 3.22E+05 | 6.62E+04 | 5.72E+04 | 3.07E+06 | 2.60E+06 | 4.91E+06 | 7.96E+05 | 1.50E+08 | 1.50E+08 |
| 2800 | 5.06E+05 | 1.06E+05 | 9.17E+04 | 4.96E+06 | 4.07E+06 | 7.66E+06 | 1.22E+06 | 1.76E+08 | 1.76E+08 |
| 2900 | 7.53E+05 | 1.60E+05 | 1.39E+05 | 7.56E+06 | 6.01E+06 | 1.13E+07 | 1.78E+06 | 2.04E+08 | 2.03E+08 |
| 3000 | 1.05E+06 | 2.27E+05 | 1.97E+05 | 1.10E+07 | 8.46E+06 | 1.57E+07 | 2.46E+06 | 2.32E+08 | 2.31E+08 |
| *T/K* | *k_9_* | *k_-9_* | *k_10_* | *k_-10_* | *k_11_* | *k_-11_* | *k_12_* | *k_-12_* | *k_13_* |
| 300 | 3.92E-05 | 2.85E-18 | 4.77E+03 | 1.62E-02 | 8.46E-05 | 4.54E-05 | 2.04E-06 | 1.74E-15 | 6.13E+01 |
| 400 | 2.17E-02 | 3.86E-14 | 4.47E+04 | 1.45E+00 | 5.60E-02 | 3.42E-02 | 2.31E-03 | 7.16E-11 | 2.80E+03 |
| 500 | 8.09E-01 | 2.12E-11 | 1.56E+05 | 2.86E+01 | 2.59E+00 | 1.78E+00 | 1.97E-01 | 8.66E-08 | 3.27E+04 |
| 600 | 7.39E+00 | 2.09E-09 | 2.82E+05 | 2.16E+02 | 3.05E+01 | 2.31E+01 | 3.84E+00 | 1.33E-05 | 1.54E+05 |
| 700 | 2.87E+01 | 6.66E-08 | 3.11E+05 | 8.54E+02 | 1.58E+02 | 1.29E+02 | 2.79E+01 | 5.14E-04 | 3.85E+05 |
| 800 | 7.33E+01 | 1.07E-06 | 2.80E+05 | 2.45E+03 | 5.50E+02 | 4.77E+02 | 1.17E+02 | 8.50E-03 | 6.99E+05 |
| 900 | 1.34E+02 | 8.99E-06 | 2.10E+05 | 5.09E+03 | 1.38E+03 | 1.24E+03 | 3.06E+02 | 6.90E-02 | 9.60E+05 |
| 1000 | 1.90E+02 | 4.26E-05 | 1.39E+05 | 8.03E+03 | 2.66E+03 | 2.47E+03 | 5.66E+02 | 3.11E-01 | 1.10E+06 |
| 1100 | 2.27E+02 | 1.24E-04 | 8.64E+04 | 1.01E+04 | 4.15E+03 | 3.92E+03 | 8.16E+02 | 8.70E-01 | 1.11E+06 |
| 1200 | 2.12E+02 | 2.20E-04 | 4.73E+04 | 9.55E+03 | 4.84E+03 | 4.62E+03 | 8.63E+02 | 1.50E+00 | 8.45E+05 |
| 1300 | 1.76E+02 | 2.88E-04 | 2.51E+04 | 7.82E+03 | 4.79E+03 | 4.61E+03 | 7.56E+02 | 1.94E+00 | 5.00E+05 |
| 1400 | 1.17E+02 | 2.65E-04 | 1.17E+04 | 5.26E+03 | 3.83E+03 | 3.71E+03 | 5.14E+02 | 1.96E+00 | 2.36E+05 |
| 1500 | 6.93E+01 | 1.97E-04 | 5.21E+03 | 3.44E+03 | 3.26E+03 | 3.19E+03 | 3.05E+02 | 2.49E+00 | 1.04E+05 |
| 1600 | 4.10E+01 | 1.39E-04 | 2.49E+03 | 2.17E+03 | 4.25E+03 | 4.21E+03 | 1.80E+02 | 3.97E+00 | 4.92E+04 |
| 1700 | 2.16E+01 | 9.37E-05 | 1.11E+03 | 1.06E+03 | 7.68E+03 | 7.66E+03 | 9.44E+01 | 2.93E+00 | 2.17E+04 |
| 1800 | 1.27E+01 | 1.04E-04 | 5.73E+02 | 5.50E+02 | 1.39E+04 | 1.38E+04 | 5.48E+01 | 1.46E+00 | 1.10E+04 |
| 1900 | 1.04E+01 | 1.70E-04 | 4.26E+02 | 4.05E+02 | 1.77E+04 | 1.77E+04 | 4.47E+01 | 9.63E-01 | 8.10E+03 |
| 2000 | 2.19E+01 | 3.91E-04 | 8.28E+02 | 7.78E+02 | 2.55E+04 | 2.55E+04 | 9.32E+01 | 1.64E+00 | 1.55E+04 |
| 2100 | 6.76E+01 | 1.08E-03 | 2.41E+03 | 2.23E+03 | 4.55E+04 | 4.54E+04 | 2.84E+02 | 4.17E+00 | 4.44E+04 |
| 2200 | 1.95E+02 | 2.71E-03 | 6.61E+03 | 6.03E+03 | 7.96E+04 | 7.94E+04 | 8.10E+02 | 1.01E+01 | 1.20E+05 |
| 2300 | 4.96E+02 | 6.05E-03 | 1.61E+04 | 1.45E+04 | 1.31E+05 | 1.30E+05 | 2.03E+03 | 2.20E+01 | 2.87E+05 |
| 2400 | 1.11E+03 | 1.21E-02 | 3.49E+04 | 3.09E+04 | 2.03E+05 | 2.02E+05 | 4.49E+03 | 4.27E+01 | 6.11E+05 |
| 2500 | 2.24E+03 | 2.19E-02 | 6.83E+04 | 5.96E+04 | 2.97E+05 | 2.95E+05 | 8.94E+03 | 7.56E+01 | 1.18E+06 |
| 2600 | 4.13E+03 | 3.66E-02 | 1.23E+05 | 1.05E+05 | 4.15E+05 | 4.11E+05 | 1.62E+04 | 1.23E+02 | 2.08E+06 |
| 2700 | 7.03E+03 | 5.72E-02 | 2.04E+05 | 1.73E+05 | 5.55E+05 | 5.48E+05 | 2.73E+04 | 1.88E+02 | 3.41E+06 |
| 2800 | 1.12E+04 | 8.40E-02 | 3.19E+05 | 2.66E+05 | 7.15E+05 | 7.04E+05 | 4.29E+04 | 2.70E+02 | 5.25E+06 |
| 2900 | 1.68E+04 | 1.17E-01 | 4.71E+05 | 3.87E+05 | 8.89E+05 | 8.72E+05 | 6.35E+04 | 3.68E+02 | 7.65E+06 |
| 3000 | 2.37E+04 | 1.55E-01 | 6.57E+05 | 5.32E+05 | 1.06E+06 | 1.04E+06 | 8.86E+04 | 4.76E+02 | 1.06E+07 |
| *T/K* | *k_-13_* | *k_14_* | *k_-14_* | *k_15_* | *k_-15_* | *k_16_* | *k_-16_* | *k_17_* | *k_-17_* |
| 300 | 5.50E-07 | 7.19E+01 | 1.06E+01 | 3.71E-05 | 3.70E-05 | 8.81E-10 | 4.29E-19 | 1.97E+07 | 9.29E+06 |
| 400 | 1.74E-04 | 3.60E+03 | 8.03E+02 | 3.81E-01 | 3.81E-01 | 3.80E-05 | 2.15E-13 | 2.76E+07 | 1.71E+07 |
| 500 | 9.07E-03 | 4.72E+04 | 1.45E+04 | 7.39E+01 | 7.38E+01 | 1.93E-02 | 6.35E-10 | 3.57E+07 | 2.56E+07 |
| 600 | 1.56E-01 | 2.57E+05 | 1.03E+05 | 1.67E+03 | 1.67E+03 | 8.58E-01 | 1.33E-07 | 4.65E+07 | 3.71E+07 |
| 700 | 1.25E+00 | 7.61E+05 | 3.76E+05 | 9.78E+03 | 9.76E+03 | 8.60E+00 | 5.97E-06 | 6.02E+07 | 5.17E+07 |
| 800 | 6.47E+00 | 1.67E+06 | 9.74E+05 | 2.69E+04 | 2.69E+04 | 3.86E+01 | 1.11E-04 | 7.66E+07 | 6.92E+07 |
| 900 | 2.24E+01 | 2.79E+06 | 1.83E+06 | 4.24E+04 | 4.23E+04 | 9.78E+01 | 9.93E-04 | 8.25E+07 | 7.71E+07 |
| 1000 | 5.58E+01 | 3.81E+06 | 2.71E+06 | 4.44E+04 | 4.42E+04 | 1.68E+02 | 4.81E-03 | 8.87E+07 | 8.48E+07 |
| 1100 | 1.06E+02 | 4.51E+06 | 3.40E+06 | 3.28E+04 | 3.26E+04 | 2.22E+02 | 1.42E-02 | 8.81E+07 | 8.54E+07 |
| 1200 | 1.40E+02 | 4.07E+06 | 3.22E+06 | 1.86E+04 | 1.84E+04 | 2.21E+02 | 2.53E-02 | 7.53E+07 | 7.37E+07 |
| 1300 | 1.49E+02 | 3.03E+06 | 2.53E+06 | 9.92E+03 | 9.73E+03 | 1.92E+02 | 3.37E-02 | 5.92E+07 | 5.83E+07 |
| 1400 | 1.17E+02 | 1.85E+06 | 1.61E+06 | 4.59E+03 | 4.46E+03 | 1.32E+02 | 3.50E-02 | 3.96E+07 | 3.92E+07 |
| 1500 | 7.77E+01 | 1.19E+06 | 1.09E+06 | 2.03E+03 | 1.95E+03 | 8.00E+01 | 4.54E-02 | 2.41E+07 | 2.39E+07 |
| 1600 | 5.05E+01 | 1.15E+06 | 1.10E+06 | 9.60E+02 | 9.12E+02 | 4.81E+01 | 6.91E-02 | 1.53E+07 | 1.52E+07 |
| 1700 | 3.25E+01 | 1.74E+06 | 1.72E+06 | 4.27E+02 | 4.02E+02 | 2.58E+01 | 4.78E-02 | 1.04E+07 | 1.04E+07 |
| 1800 | 3.76E+01 | 3.27E+06 | 3.26E+06 | 2.20E+02 | 2.04E+02 | 1.53E+01 | 2.42E-02 | 1.10E+07 | 1.10E+07 |
| 1900 | 8.49E+01 | 6.17E+06 | 6.16E+06 | 1.63E+02 | 1.50E+02 | 1.26E+01 | 1.64E-02 | 1.72E+07 | 1.72E+07 |
| 2000 | 2.30E+02 | 1.11E+07 | 1.11E+07 | 3.17E+02 | 2.90E+02 | 2.68E+01 | 2.89E-02 | 3.18E+07 | 3.18E+07 |
| 2100 | 5.73E+02 | 1.88E+07 | 1.87E+07 | 9.23E+02 | 8.39E+02 | 8.35E+01 | 7.61E-02 | 5.91E+07 | 5.90E+07 |
| 2200 | 1.27E+03 | 3.00E+07 | 2.99E+07 | 2.54E+03 | 2.30E+03 | 2.43E+02 | 1.91E-01 | 1.05E+08 | 1.05E+08 |
| 2300 | 2.54E+03 | 4.55E+07 | 4.52E+07 | 6.20E+03 | 5.58E+03 | 6.21E+02 | 4.28E-01 | 1.77E+08 | 1.77E+08 |
| 2400 | 4.59E+03 | 6.55E+07 | 6.48E+07 | 1.35E+04 | 1.21E+04 | 1.40E+03 | 8.57E-01 | 2.84E+08 | 2.84E+08 |
| 2500 | 7.64E+03 | 9.00E+07 | 8.88E+07 | 2.65E+04 | 2.36E+04 | 2.84E+03 | 1.56E+00 | 4.36E+08 | 4.36E+08 |
| 2600 | 1.19E+04 | 1.19E+08 | 1.17E+08 | 4.77E+04 | 4.24E+04 | 5.25E+03 | 2.61E+00 | 6.39E+08 | 6.39E+08 |
| 2700 | 1.73E+04 | 1.51E+08 | 1.47E+08 | 7.97E+04 | 7.07E+04 | 8.98E+03 | 4.09E+00 | 9.00E+08 | 9.00E+08 |
| 2800 | 2.39E+04 | 1.85E+08 | 1.80E+08 | 1.25E+05 | 1.11E+05 | 1.44E+04 | 6.02E+00 | 1.22E+09 | 1.22E+09 |
| 2900 | 3.17E+04 | 2.21E+08 | 2.14E+08 | 1.85E+05 | 1.63E+05 | 2.16E+04 | 8.41E+00 | 1.60E+09 | 1.60E+09 |
| 3000 | 4.02E+04 | 2.58E+08 | 2.47E+08 | 2.58E+05 | 2.27E+05 | 3.06E+04 | 1.11E+01 | 2.02E+09 | 2.02E+09 |

**Table S6.** Unimolecular rate constants (s^−1^) calculated by RRKM theory at 1 atm pressure for all forward and reverse reactions represented in scheme 1 (starting from the pre-reactive complex).

| T/K | P/bar | k1 (L mol^−1^ s^−1^) | k_2_ (L mol^−1^ s ^−1^) | k_3_ (L mol^−1^ s ^−1^) | k_4_ (L mol^−1^ s ^−1^) |
| --- | --- | --- | --- | --- | --- |
| 200 | 1.00E-04 | 8.05E+02 | 9.61E+05 | 3.44E-16 | 1.60E-33 |
| 220 | 1.00E-04 | 5.91E+02 | 4.05E+05 | 8.45E-15 | 1.83E-32 |
| 240 | 1.00E-04 | 4.73E+02 | 2.09E+05 | 1.37E-13 | 3.90E-31 |
| 260 | 1.00E-04 | 4.03E+02 | 1.25E+05 | 1.54E-12 | 1.34E-29 |
| 280 | 1.00E-04 | 3.58E+02 | 8.28E+04 | 1.28E-11 | 5.68E-28 |
| 300 | 1.00E-04 | 3.29E+02 | 5.96E+04 | 8.29E-11 | 2.24E-26 |
| 320 | 1.00E-04 | 3.09E+02 | 4.56E+04 | 4.37E-10 | 7.02E-25 |
| 340 | 1.00E-04 | 2.97E+02 | 3.66E+04 | 1.95E-09 | 1.66E-23 |
| 360 | 1.00E-04 | 2.90E+02 | 3.05E+04 | 7.55E-09 | 3.00E-22 |
| 380 | 1.00E-04 | 2.87E+02 | 2.62E+04 | 2.60E-08 | 4.23E-21 |
| 400 | 1.00E-04 | 2.89E+02 | 2.32E+04 | 8.14E-08 | 4.79E-20 |
| 420 | 1.00E-04 | 2.95E+02 | 2.10E+04 | 2.34E-07 | 4.48E-19 |
| 440 | 1.00E-04 | 3.05E+02 | 1.94E+04 | 6.28E-07 | 3.53E-18 |
| 460 | 1.00E-04 | 3.19E+02 | 1.83E+04 | 1.59E-06 | 2.40E-17 |
| 480 | 1.00E-04 | 3.38E+02 | 1.76E+04 | 3.80E-06 | 1.44E-16 |
| 500 | 1.00E-04 | 3.62E+02 | 1.71E+04 | 8.70E-06 | 7.64E-16 |
| 520 | 1.00E-04 | 3.92E+02 | 1.69E+04 | 1.91E-05 | 3.68E-15 |
| 540 | 1.00E-04 | 4.29E+02 | 1.69E+04 | 4.06E-05 | 1.61E-14 |
| 560 | 1.00E-04 | 4.75E+02 | 1.71E+04 | 8.37E-05 | 6.54E-14 |
| 580 | 1.00E-04 | 5.32E+02 | 1.76E+04 | 1.68E-04 | 2.46E-13 |
| 600 | 1.00E-04 | 6.00E+02 | 1.82E+04 | 3.27E-04 | 8.69E-13 |
| 620 | 1.00E-04 | 6.84E+02 | 1.91E+04 | 6.25E-04 | 2.89E-12 |
| 640 | 1.00E-04 | 7.85E+02 | 2.02E+04 | 1.17E-03 | 9.08E-12 |
| 660 | 1.00E-04 | 9.09E+02 | 2.16E+04 | 2.15E-03 | 2.72E-11 |
| 680 | 1.00E-04 | 8.82E+02 | 1.96E+04 | 3.21E-03 | 6.45E-11 |
| 700 | 1.00E-04 | 1.04E+03 | 2.14E+04 | 5.72E-03 | 1.78E-10 |
| 720 | 1.00E-04 | 1.24E+03 | 2.36E+04 | 1.01E-02 | 4.72E-10 |
| 740 | 1.00E-04 | 1.48E+03 | 2.63E+04 | 1.75E-02 | 1.21E-09 |
| 760 | 1.00E-04 | 1.78E+03 | 2.94E+04 | 2.99E-02 | 3.00E-09 |
| 780 | 1.00E-04 | 2.15E+03 | 3.31E+04 | 5.05E-02 | 7.21E-09 |
| 800 | 1.00E-04 | 2.60E+03 | 3.76E+04 | 8.43E-02 | 1.68E-08 |
| T/K | P/bar | k1 (L mol^−1^ s^−1^) | k_2_ (L mol^−1^ s ^−1^) | k_3_ (L mol^−1^ s ^−1^) | k_4_ (L mol^−1^ s ^−1^) |
| 200 | 1.00$E$-03 | 1.00E+03 | 1.00E+06 | 3.79E-15 | 1.26E-32 |
| 220 | 1.00$E$-03 | 7.69E+02 | 4.36E+05 | 9.17E-14 | 1.82E-31 |
| 240 | 1.00$E$-03 | 6.54E+02 | 2.32E+05 | 1.45E-12 | 4.06E-30 |
| 260 | 1.00$E$-03 | 6.00E+02 | 1.44E+05 | 1.60E-11 | 1.37E-28 |
| 280 | 1.00$E$-03 | 5.79E+02 | 1.00E+05 | 1.29E-10 | 5.66E-27 |
| 300 | 1.00$E$-03 | 5.80E+02 | 7.64E+04 | 8.12E-10 | 2.17E-25 |
| 320 | 1.00$E$-03 | 5.96E+02 | 6.21E+04 | 4.13E-09 | 6.55E-24 |
| 340 | 1.00$E$-03 | 6.21E+02 | 5.30E+04 | 1.77E-08 | 1.49E-22 |
| 360 | 1.00$E$-03 | 6.54E+02 | 4.69E+04 | 6.51E-08 | 2.57E-21 |
| 380 | 1.00$E$-03 | 6.93E+02 | 4.28E+04 | 2.12E-07 | 3.42E-20 |
| 400 | 1.00$E$-03 | 7.37E+02 | 3.98E+04 | 6.24E-07 | 3.65E-19 |
| 420 | 1.00$E$-03 | 7.86E+02 | 3.77E+04 | 1.68E-06 | 3.19E-18 |
| 440 | 1.00$E$-03 | 8.40E+02 | 3.62E+04 | 4.19E-06 | 2.34E-17 |
| 460 | 1.00$E$-03 | 9.00E+02 | 3.52E+04 | 9.81E-06 | 1.48E-16 |
| 480 | 1.00$E$-03 | 9.65E+02 | 3.46E+04 | 2.17E-05 | 8.16E-16 |
| 500 | 1.00$E$-03 | 1.04E+03 | 3.43E+04 | 4.58E-05 | 4.01E-15 |
| 520 | 1.00$E$-03 | 1.12E+03 | 3.43E+04 | 9.29E-05 | 1.78E-14 |
| 540 | 1.00$E$-03 | 1.21E+03 | 3.45E+04 | 1.82E-04 | 7.19E-14 |
| 560 | 1.00$E$-03 | 1.32E+03 | 3.50E+04 | 3.44E-04 | 2.68E-13 |
| 580 | 1.00$E$-03 | 1.44E+03 | 3.58E+04 | 6.35E-04 | 9.31E-13 |
| 600 | 1.00$E$-03 | 1.58E+03 | 3.68E+04 | 1.14E-03 | 3.03E-12 |
| 620 | 1.00$E$-03 | 1.74E+03 | 3.81E+04 | 2.02E-03 | 9.30E-12 |
| 640 | 1.00$E$-03 | 1.92E+03 | 3.97E+04 | 3.50E-03 | 2.71E-11 |
| 660 | 1.00$E$-03 | 2.14E+03 | 4.17E+04 | 5.96E-03 | 7.53E-11 |
| 680 | 1.00$E$-03 | 1.98E+03 | 3.68E+04 | 8.19E-03 | 1.64E-10 |
| 700 | 1.00$E$-03 | 2.22E+03 | 3.92E+04 | 1.36E-02 | 4.21E-10 |
| 720 | 1.00$E$-03 | 2.52E+03 | 4.20E+04 | 2.22E-02 | 1.04E-09 |
| 740 | 1.00$E$-03 | 2.87E+03 | 4.53E+04 | 3.61E-02 | 2.50E-09 |
| 760 | 1.00$E$-03 | 3.30E+03 | 4.92E+04 | 5.82E-02 | 5.84E-09 |
| 780 | 1.00$E$-03 | 3.81E+03 | 5.39E+04 | 9.30E-02 | 1.33E-08 |
| 800 | 1.00$E$-03 | 4.43E+03 | 5.94E+04 | 1.47E-01 | 2.94E-08 |
| T/K | P/bar | k1 (L mol^−1^ s^−1^) | k_2_ (L mol^−1^ s ^−1^) | k_3_ (L mol^−1^ s ^−1^) | k_4_ (L mol^−1^ s ^−1^) |
| 200 | 1.00$E$-02 | 1.08E+03 | 8.23E+05 | 2.53E-14 | 5.00E-32 |
| 220 | 1.00$E$-02 | 8.47E+02 | 3.91E+05 | 6.84E-13 | 1.23E-30 |
| 240 | 1.00$E$-02 | 7.31E+02 | 2.21E+05 | 1.15E-11 | 3.80E-29 |
| 260 | 1.00$E$-02 | 6.85E+02 | 1.44E+05 | 1.32E-10 | 1.45E-27 |
| 280 | 1.00$E$-02 | 6.77E+02 | 1.03E+05 | 1.10E-09 | 6.04E-26 |
| 300 | 1.00$E$-02 | 7.01E+02 | 8.10E+04 | 7.05E-09 | 2.28E-24 |
| 320 | 1.00$E$-02 | 7.52E+02 | 6.80E+04 | 3.65E-08 | 6.78E-23 |
| 340 | 1.00$E$-02 | 8.22E+02 | 5.98E+04 | 1.57E-07 | 1.51E-21 |
| 360 | 1.00$E$-02 | 9.17E+02 | 5.51E+04 | 5.83E-07 | 2.54E-20 |
| 380 | 1.00$E$-02 | 1.03E+03 | 5.24E+04 | 1.90E-06 | 3.32E-19 |
| 400 | 1.00$E$-02 | 1.17E+03 | 5.10E+04 | 5.52E-06 | 3.43E-18 |
| 420 | 1.00$E$-02 | 1.33E+03 | 5.05E+04 | 1.46E-05 | 2.90E-17 |
| 440 | 1.00$E$-02 | 1.51E+03 | 5.08E+04 | 3.55E-05 | 2.05E-16 |
| 460 | 1.00$E$-02 | 1.71E+03 | 5.17E+04 | 8.05E-05 | 1.24E-15 |
| 480 | 1.00$E$-02 | 1.93E+03 | 5.31E+04 | 1.72E-04 | 6.55E-15 |
| 500 | 1.00$E$-02 | 2.16E+03 | 5.44E+04 | 3.44E-04 | 3.03E-14 |
| 520 | 1.00$E$-02 | 2.43E+03 | 5.63E+04 | 6.60E-04 | 1.27E-13 |
| 540 | 1.00$E$-02 | 2.71E+03 | 5.84E+04 | 1.22E-03 | 4.81E-13 |
| 560 | 1.00$E$-02 | 3.02E+03 | 6.07E+04 | 2.16E-03 | 1.67E-12 |
| 580 | 1.00$E$-02 | 3.34E+03 | 6.32E+04 | 3.71E-03 | 5.40E-12 |
| 600 | 1.00$E$-02 | 3.71E+03 | 6.61E+04 | 6.21E-03 | 1.63E-11 |
| 620 | 1.00$E$-02 | 4.09E+03 | 6.92E+04 | 1.01E-02 | 4.63E-11 |
| 640 | 1.00$E$-02 | 4.51E+03 | 7.25E+04 | 1.62E-02 | 1.24E-10 |
| 660 | 1.00$E$-02 | 4.98E+03 | 7.62E+04 | 2.55E-02 | 3.19E-10 |
| 680 | 1.00$E$-02 | 4.55E+03 | 6.72E+04 | 3.21E-02 | 6.37E-10 |
| 700 | 1.00$E$-02 | 4.99E+03 | 7.06E+04 | 4.86E-02 | 1.49E-09 |
| 720 | 1.00$E$-02 | 5.49E+03 | 7.45E+04 | 7.28E-02 | 3.37E-09 |
| 740 | 1.00$E$-02 | 6.07E+03 | 7.92E+04 | 1.08E-01 | 7.42E-09 |
| 760 | 1.00$E$-02 | 6.73E+03 | 8.43E+04 | 1.60E-01 | 1.59E-08 |
| 780 | 1.00$E$-02 | 7.49E+03 | 9.03E+04 | 2.34E-01 | 3.31E-08 |
| 800 | 1.00$E$-02 | 8.38E+03 | 9.73E+04 | 3.43E-01 | 6.78E-08 |
| T/K | P/bar | k1 (L mol^−1^ s^−1^) | k_2_ (L mol^−1^ s ^−1^) | k_3_ (L mol^−1^ s ^−1^) | k_4_ (L mol^−1^ s ^−1^) |
| 200 | 1.00$E$-01 | 7.99E+02 | 3.69E+05 | 4.41E-14 | 8.20E-32 |
| 220 | 1.00$E$-01 | 6.88E+02 | 2.11E+05 | 1.30E-12 | 3.13E-30 |
| 240 | 1.00$E$-01 | 6.41E+02 | 1.40E+05 | 2.36E-11 | 1.62E-28 |
| 260 | 1.00$E$-01 | 6.32E+02 | 1.02E+05 | 2.89E-10 | 9.48E-27 |
| 280 | 1.00$E$-01 | 6.52E+02 | 8.12E+04 | 2.58E-09 | 5.11E-25 |
| 300 | 1.00$E$-01 | 6.97E+02 | 6.85E+04 | 1.77E-08 | 2.17E-23 |
| 320 | 1.00$E$-01 | 7.64E+02 | 6.06E+04 | 9.82E-08 | 6.75E-22 |
| 340 | 1.00$E$-01 | 8.56E+02 | 5.59E+04 | 4.55E-07 | 1.53E-20 |
| 360 | 1.00$E$-01 | 9.75E+02 | 5.32E+04 | 1.81E-06 | 2.60E-19 |
| 380 | 1.00$E$-01 | 1.12E+03 | 5.21E+04 | 6.31E-06 | 3.39E-18 |
| 400 | 1.00$E$-01 | 1.31E+03 | 5.21E+04 | 1.97E-05 | 3.50E-17 |
| 420 | 1.00$E$-01 | 1.53E+03 | 5.31E+04 | 5.58E-05 | 2.94E-16 |
| 440 | 1.00$E$-01 | 1.79E+03 | 5.48E+04 | 1.45E-04 | 2.06E-15 |
| 460 | 1.00$E$-01 | 2.10E+03 | 5.73E+04 | 3.48E-04 | 1.23E-14 |
| 480 | 1.00$E$-01 | 2.47E+03 | 6.04E+04 | 7.83E-04 | 6.37E-14 |
| 500 | 1.00$E$-01 | 2.90E+03 | 6.42E+04 | 1.66E-03 | 2.91E-13 |
| 520 | 1.00$E$-01 | 3.38E+03 | 6.85E+04 | 3.32E-03 | 1.19E-12 |
| 540 | 1.00$E$-01 | 3.94E+03 | 7.33E+04 | 6.32E-03 | 4.38E-12 |
| 560 | 1.00$E$-01 | 4.57E+03 | 7.87E+04 | 1.15E-02 | 1.48E-11 |
| 580 | 1.00$E$-01 | 5.27E+03 | 8.45E+04 | 2.02E-02 | 4.61E-11 |
| 600 | 1.00$E$-01 | 6.05E+03 | 9.08E+04 | 3.41E-02 | 1.33E-10 |
| 620 | 1.00$E$-01 | 6.91E+03 | 9.76E+04 | 5.56E-02 | 3.61E-10 |
| 640 | 1.00$E$-01 | 7.85E+03 | 1.05E+05 | 8.82E-02 | 9.23E-10 |
| 660 | 1.00$E$-01 | 8.88E+03 | 1.13E+05 | 1.36E-01 | 2.23E-09 |
| 680 | 1.00$E$-01 | 8.31E+03 | 1.01E+05 | 1.68E-01 | 4.22E-09 |
| 700 | 1.00$E$-01 | 9.27E+03 | 1.08E+05 | 2.46E-01 | 9.23E-09 |
| 720 | 1.00$E$-01 | 1.03E+04 | 1.15E+05 | 3.53E-01 | 1.94E-08 |
| 740 | 1.00$E$-01 | 1.14E+04 | 1.23E+05 | 4.98E-01 | 3.94E-08 |
| 760 | 1.00$E$-01 | 1.26E+04 | 1.32E+05 | 6.91E-01 | 7.72E-08 |
| 780 | 1.00$E$-01 | 1.40E+04 | 1.41E+05 | 9.46E-01 | 1.47E-07 |
| 800 | 1.00$E$-01 | 1.54E+04 | 1.50E+05 | 1.28E+00 | 2.73E-07 |
| T/K | P/bar | k1 (L mol^−1^ s^−1^) | k_2_ (L mol^−1^ s ^−1^) | k_3_ (L mol^−1^ s ^−1^) | k_4_ (L mol^−1^ s ^−1^) |
| 200 | 1.00E+00 | 2.60E+02 | 7.66E+04 | 4.69E-14 | 8.71E-32 |
| 220 | 1.00E+00 | 2.70E+02 | 5.38E+04 | 1.40E-12 | 3.83E-30 |
| 240 | 1.00E+00 | 2.97E+02 | 4.32E+04 | 2.60E-11 | 2.46E-28 |
| 260 | 1.00E+00 | 3.39E+02 | 3.80E+04 | 3.24E-10 | 1.85E-26 |
| 280 | 1.00E+00 | 3.95E+02 | 3.55E+04 | 2.94E-09 | 1.23E-24 |
| 300 | 1.00E+00 | 4.67E+02 | 3.45E+04 | 2.05E-08 | 6.08E-23 |
| 320 | 1.00E+00 | 5.56E+02 | 3.45E+04 | 1.16E-07 | 2.11E-21 |
| 340 | 1.00E+00 | 6.65E+02 | 3.52E+04 | 5.47E-07 | 5.21E-20 |
| 360 | 1.00E+00 | 7.99E+02 | 3.66E+04 | 2.22E-06 | 9.46E-19 |
| 380 | 1.00E+00 | 9.61E+02 | 3.84E+04 | 7.97E-06 | 1.31E-17 |
| 400 | 1.00E+00 | 1.16E+03 | 4.08E+04 | 2.56E-05 | 1.43E-16 |
| 420 | 1.00E+00 | 1.39E+03 | 4.37E+04 | 7.51E-05 | 1.26E-15 |
| 440 | 1.00E+00 | 1.68E+03 | 4.70E+04 | 2.02E-04 | 9.29E-15 |
| 460 | 1.00E+00 | 2.02E+03 | 5.09E+04 | 5.08E-04 | 5.82E-14 |
| 480 | 1.00E+00 | 2.42E+03 | 5.54E+04 | 1.19E-03 | 3.16E-13 |
| 500 | 1.00E+00 | 2.90E+03 | 6.04E+04 | 2.65E-03 | 1.51E-12 |
| 520 | 1.00E+00 | 3.46E+03 | 6.61E+04 | 5.58E-03 | 6.43E-12 |
| 540 | 1.00E+00 | 4.12E+03 | 7.24E+04 | 1.12E-02 | 2.47E-11 |
| 560 | 1.00E+00 | 4.89E+03 | 7.94E+04 | 2.15E-02 | 8.65E-11 |
| 580 | 1.00E+00 | 5.77E+03 | 8.72E+04 | 3.98E-02 | 2.78E-10 |
| 600 | 1.00E+00 | 6.79E+03 | 9.57E+04 | 7.08E-02 | 8.30E-10 |
| 620 | 1.00E+00 | 7.94E+03 | 1.05E+05 | 1.22E-01 | 2.31E-09 |
| 640 | 1.00E+00 | 9.26E+03 | 1.15E+05 | 2.03E-01 | 6.03E-09 |
| 660 | 1.00E+00 | 1.07E+04 | 1.26E+05 | 3.29E-01 | 1.48E-08 |
| 680 | 1.00E+00 | 1.04E+04 | 1.17E+05 | 4.31E-01 | 2.87E-08 |
| 700 | 1.00E+00 | 1.18E+04 | 1.27E+05 | 6.56E-01 | 6.30E-08 |
| 720 | 1.00E+00 | 1.35E+04 | 1.37E+05 | 9.76E-01 | 1.32E-07 |
| 740 | 1.00E+00 | 1.52E+04 | 1.49E+05 | 1.42E+00 | 2.66E-07 |
| 760 | 1.00E+00 | 1.72E+04 | 1.61E+05 | 2.03E+00 | 5.15E-07 |
| 780 | 1.00E+00 | 1.93E+04 | 1.74E+05 | 2.83E+00 | 9.62E-07 |
| 800 | 1.00E+00 | 2.16E+04 | 1.88E+05 | 3.90E+00 | 1.74E-06 |
| T/K | P/bar | k1 (L mol^−1^ s^−1^) | k_2_ (L mol^−1^ s ^−1^) | k_3_ (L mol^−1^ s ^−1^) | k_4_ (L mol^−1^ s ^−1^) |
| 200 | 1.00E+01 | 3.86E+01 | 9.38E+03 | 4.24E-14 | 7.39E-32 |
| 220 | 1.00E+01 | 4.60E+01 | 7.32E+03 | 1.30E-12 | 3.69E-30 |
| 240 | 1.00E+01 | 5.90E+01 | 6.68E+03 | 2.43E-11 | 2.54E-28 |
| 260 | 1.00E+01 | 7.88E+01 | 6.77E+03 | 3.07E-10 | 2.02E-26 |
| 280 | 1.00E+01 | 1.08E+02 | 7.33E+03 | 2.80E-09 | 1.39E-24 |
| 300 | 1.00E+01 | 1.48E+02 | 8.28E+03 | 1.97E-08 | 7.06E-23 |
| 320 | 1.00E+01 | 2.03E+02 | 9.59E+03 | 1.12E-07 | 2.50E-21 |
| 340 | 1.00E+01 | 2.76E+02 | 1.12E+04 | 5.28E-07 | 6.27E-20 |
| 360 | 1.00E+01 | 3.71E+02 | 1.33E+04 | 2.15E-06 | 1.15E-18 |
| 380 | 1.00E+01 | 4.93E+02 | 1.57E+04 | 7.69E-06 | 1.61E-17 |
| 400 | 1.00E+01 | 6.47E+02 | 1.85E+04 | 2.47E-05 | 1.78E-16 |
| 420 | 1.00E+01 | 8.40E+02 | 2.18E+04 | 7.24E-05 | 1.60E-15 |
| 440 | 1.00E+01 | 1.08E+03 | 2.55E+04 | 1.95E-04 | 1.20E-14 |
| 460 | 1.00E+01 | 1.37E+03 | 2.98E+04 | 4.91E-04 | 7.62E-14 |
| 480 | 1.00E+01 | 1.73E+03 | 3.46E+04 | 1.16E-03 | 4.22E-13 |
| 500 | 1.00E+01 | 2.16E+03 | 4.01E+04 | 2.59E-03 | 2.06E-12 |
| 520 | 1.00E+01 | 2.68E+03 | 4.62E+04 | 5.51E-03 | 9.03E-12 |
| 540 | 1.00E+01 | 3.29E+03 | 5.30E+04 | 1.12E-02 | 3.57E-11 |
| 560 | 1.00E+01 | 4.03E+03 | 6.07E+04 | 2.19E-02 | 1.29E-10 |
| 580 | 1.00E+01 | 4.89E+03 | 6.92E+04 | 4.11E-02 | 4.31E-10 |
| 600 | 1.00E+01 | 5.90E+03 | 7.85E+04 | 7.47E-02 | 1.33E-09 |
| 620 | 1.00E+01 | 7.07E+03 | 8.89E+04 | 1.31E-01 | 3.86E-09 |
| 640 | 1.00E+01 | 8.43E+03 | 1.00E+05 | 2.25E-01 | 1.05E-08 |
| 660 | 1.00E+01 | 9.99E+03 | 1.13E+05 | 3.73E-01 | 2.69E-08 |
| 680 | 1.00E+01 | 1.02E+04 | 1.10E+05 | 5.19E-01 | 5.62E-08 |
| 700 | 1.00E+01 | 1.18E+04 | 1.22E+05 | 8.09E-01 | 1.28E-07 |
| 720 | 1.00E+01 | 1.36E+04 | 1.34E+05 | 1.23E+00 | 2.80E-07 |
| 740 | 1.00E+01 | 1.56E+04 | 1.48E+05 | 1.84E+00 | 5.86E-07 |
| 760 | 1.00E+01 | 1.78E+04 | 1.62E+05 | 2.69E+00 | 1.18E-06 |
| 780 | 1.00E+01 | 2.03E+04 | 1.77E+05 | 3.85E+00 | 2.28E-06 |
| 800 | 1.00E+01 | 2.29E+04 | 1.94E+05 | 5.43E+00 | 4.26E-06 |
| T/K | P/bar | k1 (L mol^−1^ s^−1^) | k_2_ (L mol^−1^ s ^−1^) | k_3_ (L mol^−1^ s ^−1^) | k_4_ (L mol^−1^ s ^−1^) |
| 200 | 1.00E+02 | 4.13E+00 | 9.67E+02 | 1.92E-14 | 3.41E-32 |
| 220 | 1.00E+02 | 5.12E+00 | 7.73E+02 | 6.42E-13 | 2.48E-30 |
| 240 | 1.00E+02 | 6.92E+00 | 7.30E+02 | 1.29E-11 | 2.12E-28 |
| 260 | 1.00E+02 | 9.90E+00 | 7.76E+02 | 1.74E-10 | 1.85E-26 |
| 280 | 1.00E+02 | 1.47E+01 | 8.93E+02 | 1.68E-09 | 1.33E-24 |
| 300 | 1.00E+02 | 2.21E+01 | 1.09E+03 | 1.25E-08 | 6.84E-23 |
| 320 | 1.00E+02 | 3.36E+01 | 1.37E+03 | 7.41E-08 | 2.44E-21 |
| 340 | 1.00E+02 | 5.10E+01 | 1.77E+03 | 3.66E-07 | 6.15E-20 |
| 360 | 1.00E+02 | 7.68E+01 | 2.31E+03 | 1.55E-06 | 1.13E-18 |
| 380 | 1.00E+02 | 1.14E+02 | 3.04E+03 | 5.71E-06 | 1.59E-17 |
| 400 | 1.00E+02 | 1.68E+02 | 3.99E+03 | 1.88E-05 | 1.75E-16 |
| 420 | 1.00E+02 | 2.42E+02 | 5.21E+03 | 5.63E-05 | 1.56E-15 |
| 440 | 1.00E+02 | 3.43E+02 | 6.75E+03 | 1.54E-04 | 1.16E-14 |
| 460 | 1.00E+02 | 4.80E+02 | 8.69E+03 | 3.92E-04 | 7.33E-14 |
| 480 | 1.00E+02 | 6.59E+02 | 1.11E+04 | 9.34E-04 | 4.03E-13 |
| 500 | 1.00E+02 | 8.91E+02 | 1.40E+04 | 2.10E-03 | 1.95E-12 |
| 520 | 1.00E+02 | 1.19E+03 | 1.75E+04 | 4.47E-03 | 8.46E-12 |
| 540 | 1.00E+02 | 1.56E+03 | 2.16E+04 | 9.10E-03 | 3.32E-11 |
| 560 | 1.00E+02 | 2.03E+03 | 2.65E+04 | 1.78E-02 | 1.19E-10 |
| 580 | 1.00E+02 | 2.60E+03 | 3.23E+04 | 3.35E-02 | 3.96E-10 |
| 600 | 1.00E+02 | 3.30E+03 | 3.90E+04 | 6.10E-02 | 1.22E-09 |
| 620 | 1.00E+02 | 4.15E+03 | 4.68E+04 | 1.08E-01 | 3.54E-09 |
| 640 | 1.00E+02 | 5.17E+03 | 5.57E+04 | 1.85E-01 | 9.65E-09 |
| 660 | 1.00E+02 | 6.38E+03 | 6.58E+04 | 3.09E-01 | 2.49E-08 |
| 680 | 1.00E+02 | 7.20E+03 | 7.18E+04 | 4.63E-01 | 5.59E-08 |
| 700 | 1.00E+02 | 8.67E+03 | 8.32E+04 | 7.31E-01 | 1.29E-07 |
| 720 | 1.00E+02 | 1.04E+04 | 9.58E+04 | 1.13E+00 | 2.87E-07 |
| 740 | 1.00E+02 | 1.23E+04 | 1.10E+05 | 1.71E+00 | 6.09E-07 |
| 760 | 1.00E+02 | 1.45E+04 | 1.25E+05 | 2.53E+00 | 1.24E-06 |
| 780 | 1.00E+02 | 1.69E+04 | 1.42E+05 | 3.69E+00 | 2.45E-06 |
| 800 | 1.00E+02 | 1.97E+04 | 1.60E+05 | 5.27E+00 | 4.67E-06 |
| T/K | P/bar | k1 (L mol^−1^ s^−1^) | k_2_ (L mol^−1^ s ^−1^) | k_3_ (L mol^−1^ s ^−1^) | k_4_ (L mol^−1^ s ^−1^) |
| 200 | 1.00E+03 | 4.16E-01 | 9.70E+01 | 2.88E-15 | 6.15E-33 |
| 220 | 1.00E+03 | 5.18E-01 | 7.78E+01 | 1.02E-13 | 6.65E-31 |
| 240 | 1.00E+03 | 7.07E-01 | 7.38E+01 | 2.19E-12 | 8.37E-29 |
| 260 | 1.00E+03 | 1.02E+00 | 7.90E+01 | 3.14E-11 | 9.55E-27 |
| 280 | 1.00E+03 | 1.54E+00 | 9.19E+01 | 3.26E-10 | 7.98E-25 |
| 300 | 1.00E+03 | 2.38E+00 | 1.14E+02 | 2.60E-09 | 4.48E-23 |
| 320 | 1.00E+03 | 3.72E+00 | 1.46E+02 | 1.67E-08 | 1.69E-21 |
| 340 | 1.00E+03 | 5.84E+00 | 1.94E+02 | 8.90E-08 | 4.41E-20 |
| 360 | 1.00E+03 | 9.17E+00 | 2.62E+02 | 4.07E-07 | 8.37E-19 |
| 380 | 1.00E+03 | 1.43E+01 | 3.57E+02 | 1.63E-06 | 1.20E-17 |
| 400 | 1.00E+03 | 2.22E+01 | 4.91E+02 | 5.81E-06 | 1.35E-16 |
| 420 | 1.00E+03 | 3.41E+01 | 6.75E+02 | 1.87E-05 | 1.23E-15 |
| 440 | 1.00E+03 | 5.17E+01 | 9.27E+02 | 5.52E-05 | 9.25E-15 |
| 460 | 1.00E+03 | 7.73E+01 | 1.27E+03 | 1.50E-04 | 5.92E-14 |
| 480 | 1.00E+03 | 1.14E+02 | 1.72E+03 | 3.82E-04 | 3.28E-13 |
| 500 | 1.00E+03 | 1.66E+02 | 2.33E+03 | 9.11E-04 | 1.60E-12 |
| 520 | 1.00E+03 | 2.38E+02 | 3.11E+03 | 2.05E-03 | 6.97E-12 |
| 540 | 1.00E+03 | 3.37E+02 | 4.13E+03 | 4.39E-03 | 2.74E-11 |
| 560 | 1.00E+03 | 4.70E+02 | 5.44E+03 | 8.97E-03 | 9.85E-11 |
| 580 | 1.00E+03 | 6.47E+02 | 7.09E+03 | 1.76E-02 | 3.26E-10 |
| 600 | 1.00E+03 | 8.78E+02 | 9.15E+03 | 3.31E-02 | 1.00E-09 |
| 620 | 1.00E+03 | 1.18E+03 | 1.17E+04 | 6.02E-02 | 2.87E-09 |
| 640 | 1.00E+03 | 1.56E+03 | 1.48E+04 | 1.06E-01 | 7.76E-09 |
| 660 | 1.00E+03 | 2.04E+03 | 1.87E+04 | 1.81E-01 | 1.98E-08 |
| 680 | 1.00E+03 | 2.54E+03 | 2.25E+04 | 2.88E-01 | 4.60E-08 |
| 700 | 1.00E+03 | 3.24E+03 | 2.77E+04 | 4.65E-01 | 1.06E-07 |
| 720 | 1.00E+03 | 4.10E+03 | 3.40E+04 | 7.34E-01 | 2.35E-07 |
| 740 | 1.00E+03 | 5.15E+03 | 4.14E+04 | 1.13E+00 | 4.98E-07 |
| 760 | 1.00E+03 | 6.41E+03 | 5.01E+04 | 1.71E+00 | 1.02E-06 |
| 780 | 1.00E+03 | 7.91E+03 | 6.04E+04 | 2.54E+00 | 2.01E-06 |
| 800 | 1.00E+03 | 9.71E+03 | 7.23E+04 | 3.71E+00 | 3.85E-06 |
| T/K | P/bar | k1 (L mol^−1^ s^−1^) | k_2_ (L mol^−1^ s ^−1^) | k_3_ (L mol^−1^ s ^−1^) | k_4_ (L mol^−1^ s ^−1^) |
| 200 | 1.00E+04 | 4.16E-02 | 9.70E+00 | 3.03E-16 | 6.81E-34 |
| 220 | 1.00E+04 | 5.19E-02 | 7.78E+00 | 1.08E-14 | 8.31E-32 |
| 240 | 1.00E+04 | 7.08E-02 | 7.39E+00 | 2.35E-13 | 1.23E-29 |
| 260 | 1.00E+04 | 1.03E-01 | 7.91E+00 | 3.42E-12 | 1.61E-27 |
| 280 | 1.00E+04 | 1.55E-01 | 9.22E+00 | 3.61E-11 | 1.50E-25 |
| 300 | 1.00E+04 | 2.40E-01 | 1.14E+01 | 2.93E-10 | 9.09E-24 |
| 320 | 1.00E+04 | 3.76E-01 | 1.47E+01 | 1.92E-09 | 3.65E-22 |
| 340 | 1.00E+04 | 5.94E-01 | 1.96E+01 | 1.05E-08 | 1.01E-20 |
| 360 | 1.00E+04 | 9.38E-01 | 2.66E+01 | 4.93E-08 | 2.03E-19 |
| 380 | 1.00E+04 | 1.48E+00 | 3.65E+01 | 2.03E-07 | 3.06E-18 |
| 400 | 1.00E+04 | 2.32E+00 | 5.06E+01 | 7.50E-07 | 3.62E-17 |
| 420 | 1.00E+04 | 3.60E+00 | 7.02E+01 | 2.51E-06 | 3.46E-16 |
| 440 | 1.00E+04 | 5.54E+00 | 9.76E+01 | 7.70E-06 | 2.74E-15 |
| 460 | 1.00E+04 | 8.44E+00 | 1.35E+02 | 2.19E-05 | 1.84E-14 |
| 480 | 1.00E+04 | 1.27E+01 | 1.87E+02 | 5.82E-05 | 1.07E-13 |
| 500 | 1.00E+04 | 1.90E+01 | 2.58E+02 | 1.45E-04 | 5.47E-13 |
| 520 | 1.00E+04 | 2.79E+01 | 3.53E+02 | 3.44E-04 | 2.49E-12 |
| 540 | 1.00E+04 | 4.07E+01 | 4.80E+02 | 7.75E-04 | 1.02E-11 |
| 560 | 1.00E+04 | 5.85E+01 | 6.49E+02 | 1.67E-03 | 3.83E-11 |
| 580 | 1.00E+04 | 8.33E+01 | 8.71E+02 | 3.44E-03 | 1.32E-10 |
| 600 | 1.00E+04 | 1.17E+02 | 1.16E+03 | 6.82E-03 | 4.21E-10 |
| 620 | 1.00E+04 | 1.63E+02 | 1.54E+03 | 1.31E-02 | 1.25E-09 |
| 640 | 1.00E+04 | 2.25E+02 | 2.02E+03 | 2.42E-02 | 3.50E-09 |
| 660 | 1.00E+04 | 3.07E+02 | 2.63E+03 | 4.36E-02 | 9.23E-09 |
| 680 | 1.00E+04 | 4.01E+02 | 3.32E+03 | 7.34E-02 | 2.23E-08 |
| 700 | 1.00E+04 | 5.36E+02 | 4.27E+03 | 1.25E-01 | 5.28E-08 |
| 720 | 1.00E+04 | 7.09E+02 | 5.46E+03 | 2.06E-01 | 1.20E-07 |
| 740 | 1.00E+04 | 9.30E+02 | 6.94E+03 | 3.33E-01 | 2.60E-07 |
| 760 | 1.00E+04 | 1.21E+03 | 8.77E+03 | 5.27E-01 | 5.43E-07 |
| 780 | 1.00E+04 | 1.57E+03 | 1.10E+04 | 8.17E-01 | 1.09E-06 |
| 800 | 1.00E+04 | 2.01E+03 | 1.38E+04 | 1.24E+00 | 2.13E-06 |

**Table S7**. Pressure dependent rate constants computed at the BD(T)/6-31+g(d,p) level for all addition reactions.

1 Steinfeld, J. I., Francisco, J. S. & Hase, W. L. *Chemical Kinetics and Dynamic*. (Prentice-Hall, 1989).

2 Alvarez-Idaboy, J. R., Mora-Diez, N. & Vivier-Bunge, A. A quantum chemical and classical transition state theory explanation of negative activation energies in OH addition to substituted ethenes. *Journal of the American Chemical Society* **122**, 3715-3720 (2000).

3 Eckart, C. The Penetration of a Potential Barrier by Electrons. *Physical Review* **35**, 1303-1309, doi:10.1103/PhysRev.35.1303 (1930).

4 Bao, J. L. & Truhlar, D. G. Variational transition state theory: theoretical framework and recent developments. *Chemical Society Reviews* **46**, 7548-7596 (2017).

5 Rice, O. K. & Ramsperger, H. C. THEORIES OF UNIMOLECULAR GAS REACTIONS AT LOW PRESSURES. *Journal of the American Chemical Society* **49**, 1617-1629, doi:10.1021/ja01406a001 (1927).

6 Holbrook, K. A., Pilling, M. J. & Robertson, S. H. *Unimolecular Reactions*. 2nd ed edn, (Wiley: Chichester).

7 Tee, L. S., Gotoh, S. & Stewart, W. E. Molecular parameters for normal fluids. Lennard-Jones 12-6 Potential. *Industrial & Engineering Chemistry Fundamentals* **5**, 356-363 (1966).

8 Kee, R. *et al.* Transport: a software package for the evaluation of gas-phase, multicomponent transport properties. *Chemkin Collection* (1999).

9 Miyoshi, A. *Gaussian Post Processor (GPOP)*. (University of Tokyo, 2010).

10 Miyoshi, A. *Steady-State Unimolecular Master-Equation Solver (SSUMES)*. (University of Tokyo, 2010).
